# Burden of thyroid cancer in North Africa and Middle East 1990–2019

**DOI:** 10.3389/fonc.2022.955358

**Published:** 2022-09-23

**Authors:** Seyed Aria Nejadghaderi, Sahar Saeedi Moghaddam, Sina Azadnajafabad, Negar Rezaei, Nazila Rezaei, Seyed Mohammad Tavangar, Hamidreza Jamshidi, Ali H. Mokdad, Mohsen Naghavi, Farshad Farzadfar, Bagher Larijani, GBD 2019 NAME Thyroid Cancer Collaborators, Seyed Aria Nejadghaderi

**Affiliations:** ^1^ Non-Communicable Diseases Research Center, Endocrinology and Metabolism Population Sciences Institute, Tehran University of Medical Sciences, Tehran, Iran; ^2^ Endocrinology and Metabolism Research Center, Endocrinology and Metabolism Clinical Sciences Institute, Tehran University of Medical Sciences, Tehran, Iran; ^3^ Department of Pathology, Shariati Hospital, Tehran University of Medical Sciences, Tehran, Iran; ^4^ Department of Pharmacology, School of Medicine, Shahid Beheshti University of Medical Sciences, Tehran, Iran; ^5^ Institute for Health Metrics and Evaluation, University of Washington, Seattle, WA, United States; ^6^ Department of Health Metrics Sciences, School of Medicine, University of Washington, Seattle, WA, United States

**Keywords:** north africa and middle east, thyroid cancer, incidence, mortality, disability-adjusted life years, body mass index, risk factor

## Abstract

**Background:**

Thyroid cancer is the leading cause of mortality and morbidity among cancers of the endocrine system. We aimed to describe the trends of thyroid cancer burden in North Africa and Middle East for 1990–2019.

**Methods:**

Data on burden of thyroid cancer in North Africa and Middle East from 1990 to 2019 were obtained from the Global Burden of Disease (GBD) Study 2019. Decomposition analysis was used to estimate the effects of population growth, aging, and change in incident numbers on overall change of thyroid cancer incidence. Also, we used the comparative risk assessment framework of GBD to determine the burden of thyroid cancer attributable to a high body mass index (BMI).

**Results:**

In 2019, the age-standardized incidence rate (ASIR) and age-standardized mortality rate (ASMR) of thyroid cancer were 3.5 (2.9–4) and 0.5 (0.5–0.7) per 100,000, respectively. The highest age-standardized incidence, deaths, and disability-adjusted life year (DALY) rate were in Lebanon, Afghanistan, and United Arab Emirates, respectively. The ASIR of thyroid cancer in region was about 2.5 times higher among women, which had a positive association with increasing age. In 2019, the age-standardized deaths attributable to a high BMI was 16.7% of all deaths due to thyroid cancer. In 1990–2019, the overall change in thyroid cancer incident cases was a 396% increase which was mostly driven by the increase in disease-specific incidence rate (256.8%).

**Conclusions:**

Women, the elderly above about 60 years old, and countries with a higher sociodemographic index showed higher incidence rates of thyroid cancer. Regarding our findings, it is recommended to establish preventive plans by modification in life style like weight reduction programs.

## Introduction

Thyroid cancer with an estimate of about 3.0% of new cases was the ninth cancer for incidence and the most common type of cancer of the endocrine system in 2020, globally (1). The age-standardized mortality rate (ASMR) of thyroid cancer was 0.5 per 100,000 among women and 0.3 per 100,000 among men by 2020 (1). Northern America and Western Africa had the highest and lowest age-standardized incidence rate (ASIR) in 2020, respectively (1). By 2040, thyroid cancer will be estimated as the top four estimated cancer in adults aged 20 to 49 years for both sexes (2, 3). In 2019, the greatest mortality of thyroid cancer was in the population of 70 to 74 years of age among both sexes (4). Also, the all-age disability-adjusted life years (DALYs) for thyroid cancer was 1,230,000 among both sexes in 2019, worldwide (3).

In 2019, North Africa and Middle East had an ASIR of 3.5, ASMR of 0.5, and age-standardized DALY rate of 14.9 per 100,000 population, all of which showed an increase between 1990 and 2019 (5). A health system evaluation in the North Africa and Middle East (NAME) region showed that health system outcomes are heterogeneous. Moreover, people from this region may be at a higher risk for thyroid cancer-related risk factors compared to others (6). Therefore, reporting the burden of thyroid cancer in the region could help health policymakers for resource allocation and disease control and preventive programs (7).

Few studies have been conducted on the epidemiology and burden of thyroid cancer in the NAME region, especially in North African countries, and most of them reported limitations in data registry systems and the importance for a comprehensive evaluation of burden and trend of thyroid cancer (8–11). Therefore, we aimed to use the estimates of the Global Burden of Disease (GBD) Study 2019 to report the burden of thyroid cancer and its attributable risk factor in 21 countries of NAME, by age, sex, and sociodemographic index (SDI).

## Methods

### Overview

The GBD study 2019 estimated the incidence, prevalence, deaths, years of life lost (YLLs), years lived with disability (YLDs), and DALYs of 369 diseases and injuries for 204 countries and territories, the estimations of which we used for thyroid cancer in 21 countries of the NAME GBD super-region. The NAME region is such an important region because it is one out of seven GBD super-regions in addition to one of the 21 GBD regions. The countries located in this region are Afghanistan, Algeria, Bahrain, Egypt, Iran (Islamic Republic of), Iraq, Jordan, Kuwait, Lebanon, Libya, Morocco, Oman, Palestine, Qatar, Saudi Arabia, Sudan, the Syrian Arab Republic, Tunisia, Turkey, the United Arab Emirates, and Yemen. The methods for data collection and data resources have been expressed in detail elsewhere (3). Also, this study complies with the Guidelines for Accurate and Transparent Health Estimates Reporting (GATHER) (12).

### Case definition and data sources

The classification of diseases was based on the International Classification of Diseases (ICD) and Related Health Problems version 10th which mapped to the GBD code for thyroid cancer (B.1.23) (13). We included the following ICD-10 revision codes: C73-C73.9 (malignant neoplasms of thyroid gland), D09.3 (carcinoma *in situ*: thyroid and other endocrine glands), D09.8 (carcinoma *in situ* of other specified sites), D34-D34.9 (benign neoplasm of thyroid gland), and D44.0 (neoplasm of uncertain or unknown behavior: thyroid gland) (13).

### Definitions

The SDI is “the geometric mean of 0 to 1 indices including total fertility rate under the age of 25 (TFU25), mean education for those ages 15 and older (EDU15+), and lag distributed income (LDI) per capita” (14). Countries were categorized into five quintiles based on SDI: low-SDI, low-middle-SDI, middle-SDI, high-middle-SDI, and high-SDI quintile (14). Because of the inclusion criteria for risk–outcome pairs in GBD methods which needed not to have a p-value greater than 0.1, only high BMI was included as a risk factor for thyroid cancer (15). The definition of high BMI was based on BMI >25 kg/m^2^ in adults aged ≥20 years in addition to overweight or obese children aged 1 to 19 based on International Obesity Task Force standards (15, 16).

### Statistical analysis

Decomposition analysis was implemented to assess the contribution of change in incidence rate, population growth, and population age on overall change of thyroid cancer incidence rate. This method had two steps: first, hypothetical data were created by applying age and sex structure of the population in addition to incidence rate of 1990 to the total population of the year 2019. Population growth was attributable to the difference between mentioned hypothetical data and new cases in 1990. Second, hypothetical data were created by applying incidence rate from 1990 to age and sex structure and population in 2019. The difference between the second hypothetical data and new cases in 2019 was attributable to incidence rate change. Consequently, the difference between these two hypothetical data was considered as attribution to aging of the population (17). Moreover, the burden attributable to thyroid cancer was provided by health access and quality of care index (HAQI) (18).

Also, GBD used a comparative risk assessment (CRA) framework to compare the burden attributed to each risk factor. It has been established since 2002 (19, 20), and the last updated version with details was for 2019 (15). Briefly, GBD CRA uses hierarchy of a wide range of risk factors of interests, which is updated for new risk factors by systematic review and meta-analysis for each new GBD version. High BMI was included in the analysis as a risk factor of thyroid cancer, and deaths and DALY number and rates attributable to high BMI were calculated.

We utilized R programming software version 3.6.1 (R Foundation for Statistical Computing, Vienna, Austria) for conducting statistical analysis and preparing visualizations. The 95% uncertainty intervals (UIs) were estimated through the variations in individual and aggregated data which are presented for each point estimate. The reported age-standardized rates are based on per 100,000 population. Results of the present study using data visualization tool are available online at https://vizhub.healthdata.org/gbd-compare/.

## Results

### Regional burden and trend of thyroid cancer

Thyroid cancer was responsible for 3,882 incident cases and 923 deaths in NAME among both sexes in 1990. In 2019, incident and death numbers due to thyroid cancer were 19,253 and 2,290 in this region, respectively. Moreover, the DALYs attributed to thyroid cancer increased from 30,324 in 1990 to 74,180 in 2019 in both sexes (**Table 1**).

**Table 1:** Incidence, deaths, and disability-adjusted life year (DALY) number and rate of thyroid cancer in North Africa and Middle East regions among both sexes in 1990 and 2019, by country.

Location	1990						2019					
	Incidence		Deaths		DALYs		Incidence		Deaths		DALYs	
	Number	Rate	Number	Rate	Number	Rate	Number	Rate	Number	Rate	Number	Rate
**North Africa and Middle East**	**3,882 (3,233 to 4,451)**	**1.7 (1.4 to 2)**	**923 (753 to 1,188)**	**0.5 (0.4 to 0.7)**	**30,324 (25,430 to 35,353)**	**14.4 (11.8 to 18)**	**19,253 (15,675 to 22,281)**	**3.5 (2.9 to 4)**	**2,290 (1,981 to 2,669)**	**0.5 (0.5 to 0.7)**	**74,180 (62,526 to 86,119)**	**14.9 (12.8 to 17.1)**
Afghanistan	122 (62 to 199)	1.6 (0.8 to 2.6)	56 (34 to 84)	0.8 (0.5 to 1.2)	1,769 (966 to 2774)	22.7 (12.5 to 35.5)	439 (221 to 726)	2.2 (1.3 to 3.4)	118 (75 to 173)	0.9 (0.6 to 1.2)	4,229 (2421 to 6523)	24.2 (15.2 to 35.4)
Algeria	374 (278 to 490)	2.2 (1.7 to 2.8)	61 (48 to 85)	0.5 (0.4 to 0.8)	2,196 (1718 to 2810)	14 (11 to 18.8)	1,827 (1214 to 2508)	4.3 (3 to 5.8)	172 (136 to 214)	0.5 (0.4 to 0.7)	5,795 (4300 to 7459)	15.1 (11.7 to 19.1)
Bahrain	5 (4 to 6)	2 (1.7 to 2.5)	1 (1 to 1)	0.8 (0.7 to 1)	33 (27 to 42)	17.1 (14.1 to 20.5)	45 (34 to 58)	3.4 (2.6 to 4.3)	5 (3 to 6)	0.7 (0.5 to 0.9)	142 (109 to 183)	14.6 (10.4 to 18.6)
Egypt	574 (466 to 707)	1.3 (1.1 to 1.6)	130 (111 to 190)	0.4 (0.3 to 0.7)	5,236 (4424 to 6342)	12.5 (10.7 to 17)	2,008 (1406 to 2720)	2.3 (1.7 to 3.2)	271 (186 to 435)	0.4 (0.3 to 0.7)	9,697 (6858 to 13444)	12.4 (8.6 to 18.4)
Iran (Islamic Republic of)	539 (450 to 639)	1.6 (1.3 to 1.9)	99 (86 to 118)	0.4 (0.4 to 0.5)	3,172 (2747 to 3697)	10.4 (9 to 12.3)	3,198 (1999 to 3649)	3.6 (2.3 to 4.1)	341 (245 to 375)	0.5 (0.4 to 0.5)	10,469 (6928 to 11742)	13.2 (8.9 to 14.6)
Iraq	189 (140 to 252)	1.8 (1.4 to 2.5)	43 (33 to 61)	0.5 (0.4 to 0.8)	1,400 (1081 to 1860)	14.7 (11.4 to 20.5)	1,282 (895 to 1779)	4 (2.8 to 5.5)	159 (120 to 201)	0.7 (0.5 to 0.9)	5,290 (3807 to 6979)	19.1 (14.1 to 24.6)
Jordan	57 (43 to 74)	2.8 (2.2 to 3.6)	11 (9 to 13)	0.8 (0.7 to 1)	356 (287 to 437)	20.6 (16.9 to 25.7)	321 (249 to 417)	3.6 (2.8 to 4.7)	34 (28 to 43)	0.6 (0.5 to 0.7)	1,084 (877 to 1371)	14.4 (11.7 to 18.2)
Kuwait	37 (32 to 42)	3.4 (3 to 3.8)	4 (3 to 4)	0.7 (0.6 to 0.8)	129 (114 to 145)	16.1 (13.9 to 18)	149 (120 to 186)	3.8 (3.1 to 4.7)	12 (10 to 14)	0.6 (0.5 to 0.7)	372 (303 to 459)	12.7 (10.4 to 15.3)
Lebanon	86 (63 to 113)	3.3 (2.5 to 4.3)	15 (12 to 21)	0.7 (0.6 to 1)	480 (375 to 603)	19.2 (15.2 to 24.5)	431 (306 to 582)	7.9 (5.7 to 10.8)	35 (28 to 46)	0.7 (0.5 to 0.9)	1,070 (817 to 1377)	20.2 (15.5 to 25.9)
Libya	58 (40 to 79)	2.2 (1.6 to 2.9)	9 (7 to 12)	0.5 (0.4 to 0.7)	315 (234 to 408)	13.4 (10 to 17.6)	258 (170 to 364)	3.6 (2.5 to 5)	25 (18 to 33)	0.5 (0.4 to 0.7)	857 (614 to 1166)	13.9 (10.2 to 18.7)
Morocco	390 (286 to 501)	2.1 (1.7 to 2.7)	99 (76 to 122)	0.7 (0.5 to 0.9)	3,341 (2629 to 4046)	19.7 (15.5 to 23.9)	1,462 (1013 to 2109)	4 (2.8 to 5.7)	226 (168 to 301)	0.7 (0.6 to 1)	7,099 (5234 to 9473)	20.7 (15.5 to 27.6)
Oman	16 (12 to 22)	1.5 (1.1 to 2.1)	3 (2 to 4)	0.4 (0.3 to 0.6)	93 (69 to 125)	10.6 (7.8 to 14.3)	112 (82 to 146)	3.3 (2.5 to 4)	7 (6 to 9)	0.5 (0.4 to 0.6)	284 (216 to 359)	11.8 (9.7 to 14)
Palestine	25 (17 to 36)	2.3 (1.6 to 3.3)	6 (4 to 8)	0.7 (0.5 to 1)	166 (116 to 226)	17.1 (12 to 23.1)	110 (77 to 139)	3.4 (2.4 to 4.3)	15 (11 to 18)	0.7 (0.5 to 0.9)	444 (316 to 548)	16.6 (11.8 to 20.3)
Qatar	4 (3 to 5)	1.8 (1.4 to 2.2)	1 (0 to 1)	0.7 (0.6 to 0.9)	21 (17 to 27)	14.2 (11.5 to 17.8)	59 (41 to 85)	2.9 (2.2 to 4)	3 (2 to 5)	0.6 (0.4 to 0.7)	137 (99 to 194)	11.5 (8.7 to 15.2)
Saudi Arabia	136 (97 to 196)	1.4 (1 to 2.2)	29 (21 to 46)	0.5 (0.4 to 0.9)	963 (687 to 1417)	12.2 (8.9 to 19.7)	2,259 (1528 to 3157)	6 (4.4 to 7.9)	121 (93 to 154)	0.7 (0.5 to 0.8)	5,380 (3862 to 7281)	18.9 (14.5 to 24)
Sudan	128 (77 to 189)	1.1 (0.7 to 1.6)	43 (29 to 60)	0.5 (0.3 to 0.6)	1,359 (882 to 1935)	12.2 (8 to 17.2)	569 (354 to 853)	2.1 (1.4 to 3.1)	102 (71 to 135)	0.5 (0.4 to 0.7)	3,335 (2208 to 4667)	14.3 (9.8 to 19.5)
Syrian Arab Republic	37 (26 to 48)	0.5 (0.3 to 0.7)	9 (6 to 12)	0.2 (0.1 to 0.2)	297 (207 to 381)	4.6 (3 to 6)	138 (89 to 190)	1 (0.6 to 1.3)	21 (13 to 28)	0.2 (0.1 to 0.2)	619 (396 to 840)	4.6 (3 to 6.3)
Tunisia	116 (91 to 147)	1.8 (1.4 to 2.3)	20 (16 to 26)	0.4 (0.3 to 0.5)	629 (514 to 783)	10.7 (8.7 to 13.4)	494 (337 to 717)	3.8 (2.6 to 5.4)	49 (36 to 68)	0.4 (0.3 to 0.6)	1,497 (1064 to 2055)	11.7 (8.3 to 16)
Turkey	892 (650 to 1138)	2.1 (1.5 to 2.7)	257 (196 to 316)	0.7 (0.6 to 0.9)	7,459 (5589 to 9182)	18.7 (14.2 to 23)	3,271 (2493 to 4322)	3.5 (2.7 to 4.7)	455 (353 to 635)	0.5 (0.4 to 0.8)	12,071 (9272 to 16054)	13.5 (10.3 to 17.9)
United Arab Emirates	24 (15 to 33)	2.2 (1.2 to 3.1)	4 (2 to 5)	0.8 (0.3 to 1.2)	157 (99 to 222)	19.7 (9.7 to 28.3)	444 (229 to 710)	3.9 (2.1 to 5.9)	42 (22 to 66)	0.8 (0.4 to 1.2)	1,873 (966 to 2948)	22.6 (11.7 to 34.2)
Yemen	69 (41 to 108)	1.1 (0.7 to 1.6)	23 (16 to 34)	0.5 (0.3 to 0.7)	734 (475 to 1090)	12.4 (8.3 to 17.9)	356 (228 to 517)	1.9 (1.3 to 2.7)	72 (52 to 97)	0.5 (0.4 to 0.7)	2,359 (1630 to 3283)	14.2 (10.1 to 19.2)

Data in parentheses are 95% uncertainty intervals (95% UIs); Data under the column “Rate” are age-standardized rates per 100,000; DALYs, disability-adjusted life years. The bold values in this table illustrate the regional values.

**Figure 1:**
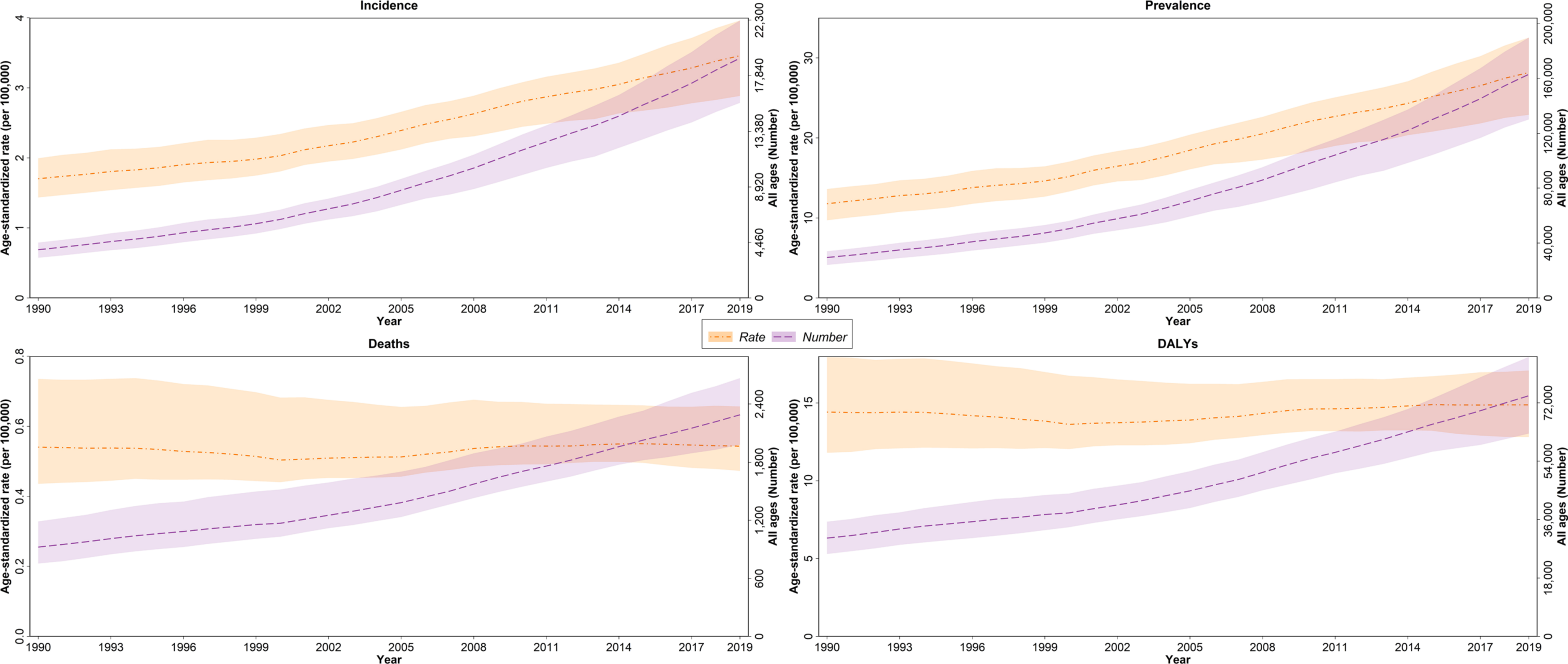
Trend of incidence, prevalence, deaths, and disability-adjusted life years (DALYs) of thyroid cancer in North Africa and Middle East regions over 1990–2019, age-standardized and for all ages.

There was a steep increase in the trend of age-standardized incidence and prevalence rate of thyroid cancer between 1990 and 2019 in NAME in both sexes, while the age-standardized rate of deaths and DALYs showed almost a steady pattern in this period (**Figure 1**).

### National burden and trend of thyroid cancer

**Figure 2:**
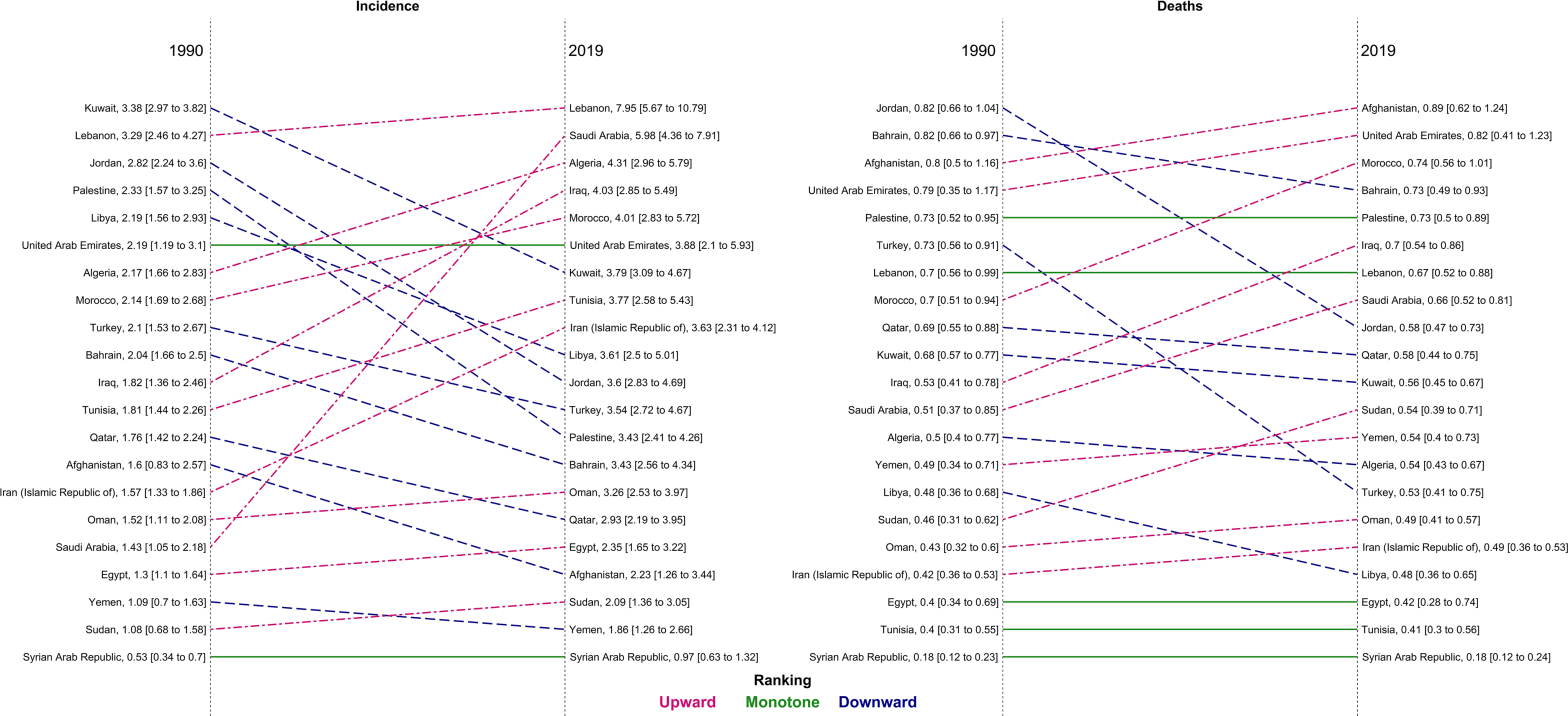
Comparison of the age-standardized rate of incidence and deaths of thyroid cancer in North Africa and Middle East regions for both sexes between 1990 and 2019, by country.

In 1990, the highest three countries in ASIR in region were Kuwait (3.4), Lebanon (3.3), and Jordan (2.8) (**Supplementary Figure 1**). In 2019, Lebanon reached to the top country with the highest ASIR and Saudi Arabia and Algeria got the second and third countries in incidence, respectively (**Table 1**; **Figure 2**; **Supplementary Figure 2**). Afghanistan and United Arab Emirates (UAE) had the highest age-standardized deaths and DALY rate in 2019 among both sexes (ASMR: 0.9 in Afghanistan and 0.8 in UAE; age-standardized DALY rate: 24.2 in Afghanistan and 22.6 in UAE). However, Syrian Arab Republic had the lowest age-standardized death and DALY rate (**Table 1** and **Figure 2**).

Saudi Arabia and Lebanon had the highest increase in incidence rate of thyroid cancer (**Figure 2**). Most countries, however, had a steady age-standardized rate of deaths and DALYs between 1990 and 2019 except for Bahrain and UAE which experienced a peak in the 2000s (**Supplementary Figure 3**).

### Burden of thyroid cancer by sex

The ASIR of thyroid cancer in women increased from 2.6 in 1990 to 5.1 in 2019 in addition to men that showed a growth from 0.8 to 2.0 between 1990 and 2019 (**Supplementary Table 1**). The ASMR and age-standardized DALY rate decreased in women over 1990–2019. Regarding men, there were no changes in ASMRs; however, an increase in age-standardized DALY rates happened since 1990. Nevertheless, there was not a significant difference between men and women in terms of ASMRs and age-standardized DALY rates in 1990 and 2019 (**Supplementary Table 1**).

### Burden of thyroid cancer by age

**Figure 3:**
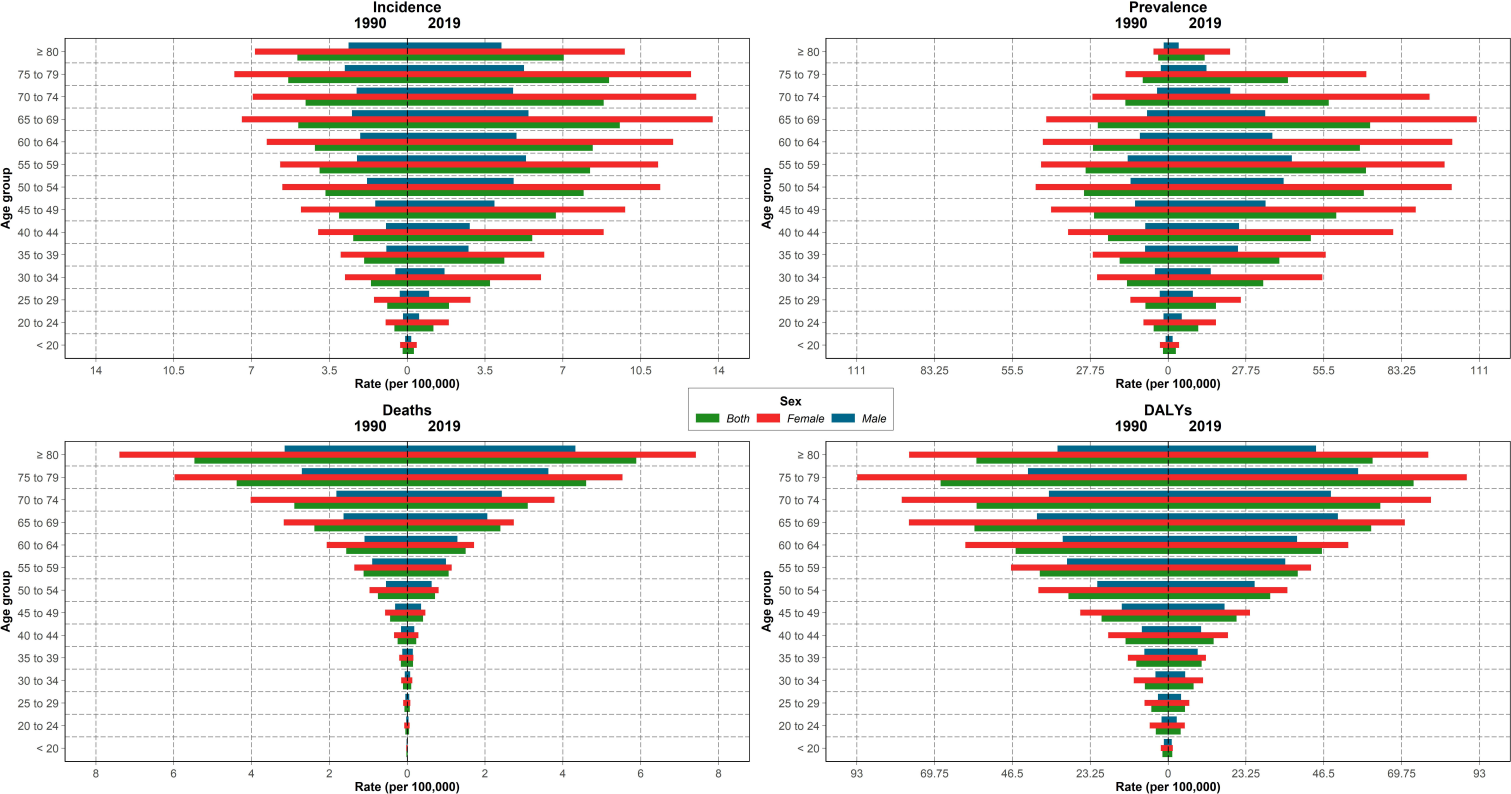
Incidence, prevalence, death, and disability-adjusted life year (DALY) rate of thyroid cancer in North Africa and Middle East regions in 1990 and 2019, by sex and age.

The incidence and prevalence rate of thyroid cancer of both sexes in the NAME region increased from birth up to the seventh decade of life and then decreased, whereas the rate of deaths and DALY in both sexes almost elevated by increasing the age (**Figure 3**). Women aged 65–69 years had the highest incidence and prevalence in 2019, while the highest prevalence in men were in the age group of 55–59 years. The highest rates of deaths and DALYs in men and women were in ≥80 and 75–79 years of age, respectively (**Figure 3**).

### High BMI-attributable thyroid cancer burden

**Figure 4:**
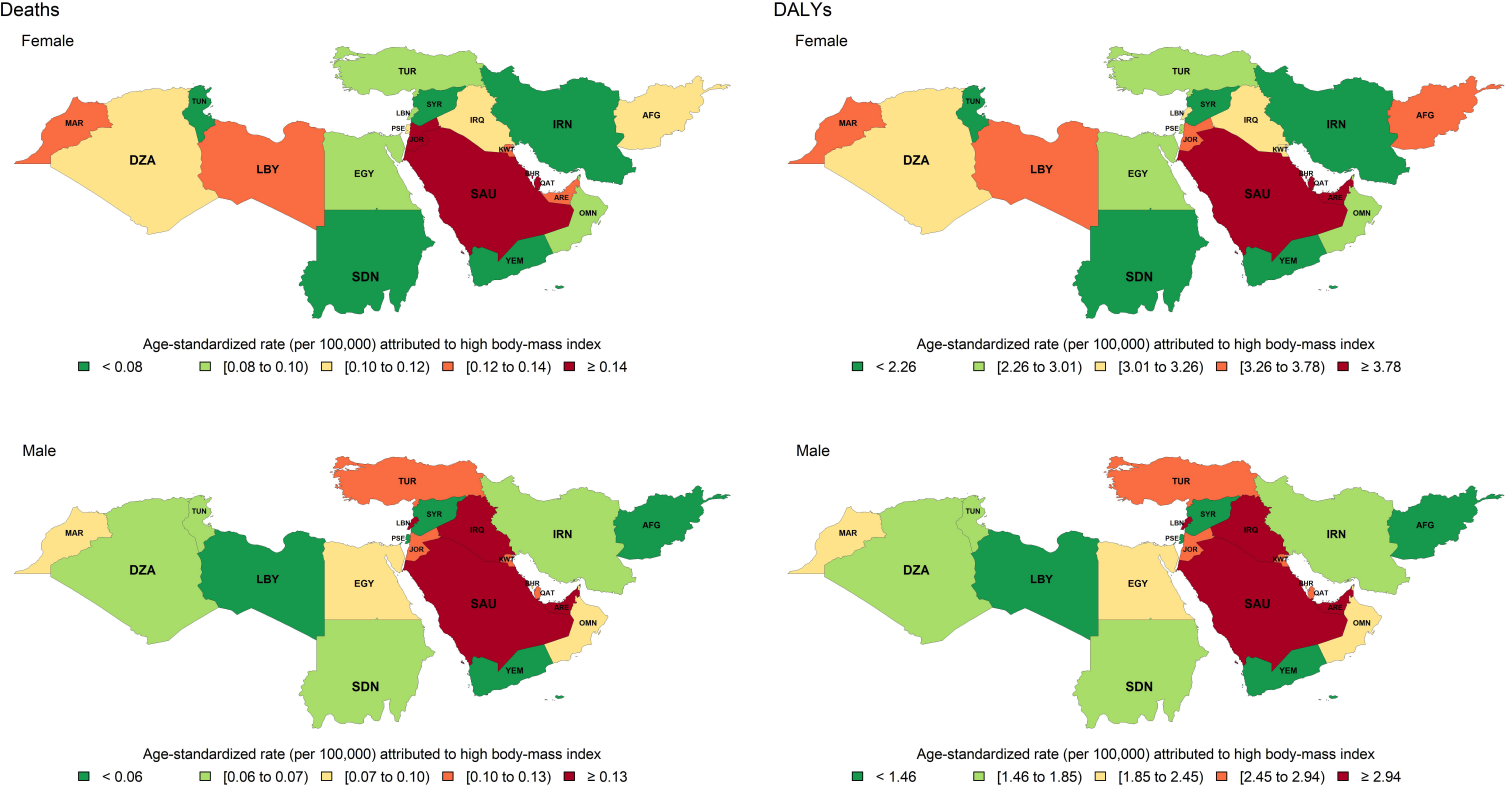
Age-standardized rate of death and disability-adjusted life years (DALYs) of thyroid cancer attributable to high body mass index (BMI) in North Africa and Middle East region in 2019, by sex and country. AFG, Afghanistan; DZA, Algeria; BHR, Bahrain; EGY, Egypt; IRN: Iran (Islamic Republic of); IRQ, Iraq; JOR, Jordan; KWT, Kuwait; LBN, Lebanon; LBY, Libya; MAR, Morocco; OMN, Oman; PSE, Palestine; QAT, Qatar; SAU, Saudi Arabia; SDN, Sudan; SYR, Syrian Arab Republic; TUN, Tunisia; TUR, Turkey; ARE, United Arab Emirates; YEM, Yemen.

The percentage of DALYs and deaths of thyroid cancer attributable to high BMI to total burden increased from 11.3% to 17.1% and from 11.2% to 16.7% between 1990 and 2019, respectively (**Supplementary Table 2**; **Supplementary Figures 4**, **5**). In 2019, Saudi Arabia and Qatar had the highest age-standardized deaths and DALY rate attributable to high BMI in women. However, not only Saudi Arabia had high age-standard deaths and DALY rate attributable to high BMI in men, but also Lebanon, Iraq, and UAE were among countries with the highest rate (**Figure 4**).

### Regional and national burden of thyroid cancer by SDI

**Figure 5:**
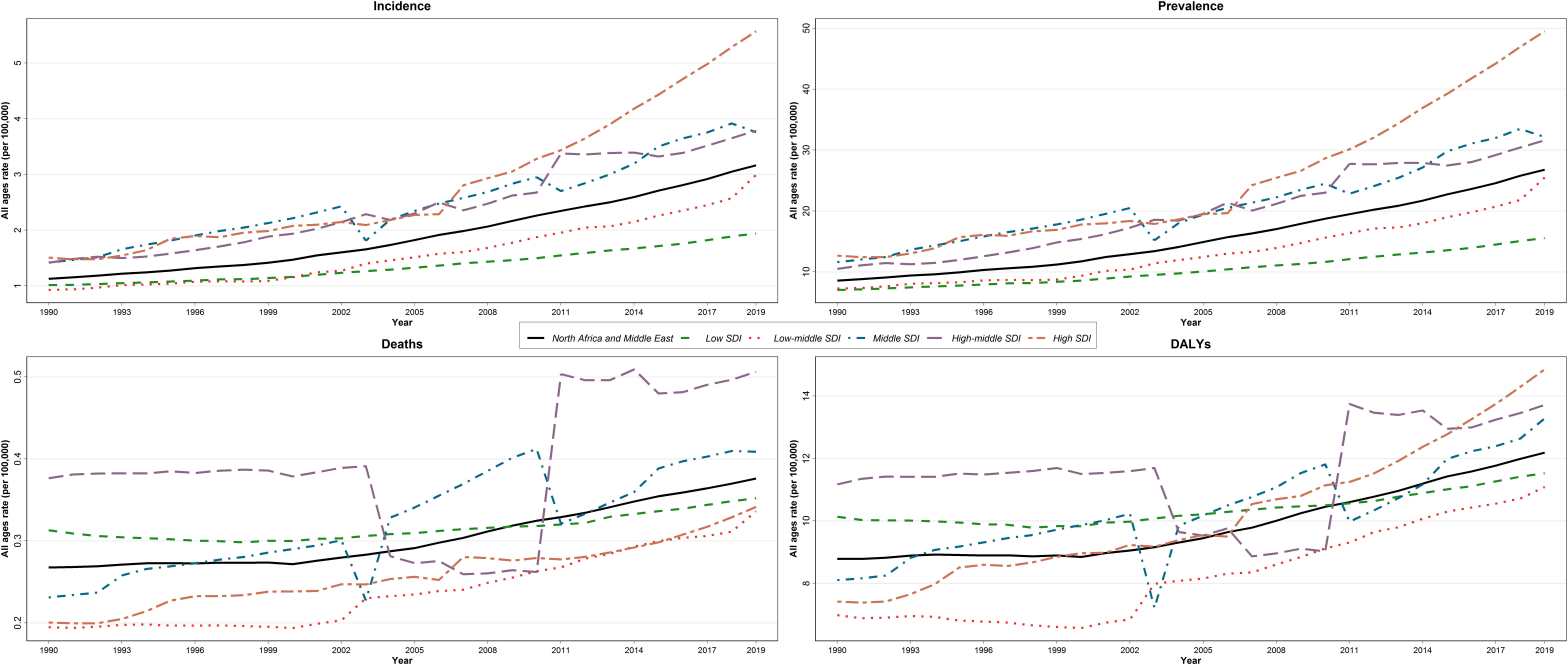
Trends of the rate of incidence, prevalence, deaths, and disability-adjusted life years (DALYs) of thyroid cancer in North Africa and Middle East regions among both sexes over 1990–2019, by sociodemographic index (SDI).

The incidence and prevalence rate of thyroid cancer increased in all SDI quintiles over 1990–2019, although it was with a greater slope in the high-SDI quintile (**Figure 5**). There was a fall in mortality and DALY rate of thyroid cancer in the middle- and high-middle-SDI quintiles in the early 2000s and a steep increase in the next years. However, low-, low-middle- and high-SDI quintiles had an increase in mortality and DALY rates with a slow slope. In 2019, high- and high-middle-SDI quintiles had the greatest rate of deaths and DALYs (**Figure 5**). The age-standardized incidence, death, and DALY rates by HAQI in 1990 and 2019 are presented in **Supplementary Figures 6**–**8**, respectively.

### Decomposition analysis

Between 1990 and 2019, the overall percent of new thyroid cancer cases was 396% in the NAME region among both sexes, which was mostly due to a change in incidence rate (256.8%) (**Table 2**). Population growth and population aging were responsible for the 76.4% and 62.7% increase in incident cases in this region, respectively, ranging from 12.4% in the Syrian Arab Republic to 543.5% in Qatar for population growth and from -68.4% in Afghanistan to 338.0% in UAE for population aging. On the other hand, a change in incidence rate which had the greatest contribution to the increasing incidence of thyroid cancer had a range from 30.9% in Kuwait to 1318.7% in Saudi Arabia in 2019 (**Table 2**). The change in incidence rate among men was much higher than among women (377.1% vs. 223.1%). Also, the overall change in percentage of new cases over 1990–2019 was 521.8% in men and 357.4% in women, and the male population in UAE and women in Egypt with an overall change of 2464.0% and 206.6% had the highest and lowest percent in overall change of new cases, respectively (**Supplementary Table 3**).

**Table 2:** Decomposition analysis of thyroid cancer incidence among both sexes in North Africa and Middle East region over 1990–2019, by country.

Location	New cases		Expected new cases in 2019		% 1990–2019 new cases change cause			% 1990–2019 new cases overall change
	Year		Given population growth alone	Given population growth and aging	Due to population growth	Due to population aging	Due to change in incidence rates	
	1990	2019						
**North Africa and Middle East**	**3,882**	**19,253**	**6,848**	**9,284**	**76.4%**	**62.7%**	**256.8%**	**396%**
Afghanistan	122	439	410	327	235.2%	-68.4%	92.3%	259.1%
Algeria	374	1,827	619	909	65.5%	77.3%	245.5%	388.3%
Bahrain	5	45	14	26	184.0%	258.3%	386.9%	829.2%
Egypt	574	2,008	1,021	1,142	77.9%	21.1%	150.8%	249.8%
Iran (Islamic Republic of)	539	3,198	776	1,353	44.0%	106.9%	342.3%	493.1%
Iraq	189	1,282	453	589	139.4%	72.1%	366.9%	578.3%
Jordan	57	321	176	256	208.4%	140.5%	113.5%	462.5%
Kuwait	37	149	92	138	151.6%	124.2%	30.9%	306.7%
Lebanon	86	431	136	178	58.1%	49.2%	293.6%	400.8%
Libya	58	258	93	154	59.0%	104.6%	179.1%	342.6%
Morocco	390	1,462	555	769	42.1%	54.9%	177.4%	274.5%
Oman	16	112	38	51	135.9%	79.3%	368.7%	583.8%
Palestine	25	110	60	74	139.4%	54.0%	145.6%	339.1%
Qatar	4	59	25	32	543.5%	192.7%	677.7%	1413.9%
Saudi Arabia	136	2,259	303	465	122.7%	118.7%	1318.7%	1560.2%
Sudan	128	569	258	282	102.0%	19.0%	225.1%	346.1%
Syrian Arab Republic	37	138	41	74	12.4%	88.3%	174.1%	274.8%
Tunisia	116	494	159	236	37.1%	66.3%	223.6%	327.0%
Turkey	892	3,271	1,215	1,941	36.1%	81.4%	148.9%	266.5%
United Arab Emirates	24	444	116	195	393.7%	338.0%	1058.5%	1790.2%
Yemen	69	356	159	196	129.5%	53.3%	231.4%	414.1%

The bold values in this table illustrate the regional values.

## Discussion

In this study, we reported the burden of thyroid cancer in an over 30-year period in the NAME region. The ASIR of thyroid cancer in both sexes almost doubled over 1990–2019, while there was no growth in ASMR within this period. Also, deaths and DALYs attributable to high BMI increased from almost 11% to 17% between 1990 and 2019. According to our decomposition analysis, the overall change in incidence was 396% over 1990–2019 and ranged from 249.8% to 1790.2% in Egypt and UAE, respectively.

We revealed that the regional incidence rate of thyroid cancer despite its deaths and DALYs is increasing. The potential reason suggested for the global increase in incidence of thyroid cancer which has been shown in literature in recent years is diagnosis of subclinical thyroid lesions due to global improvement of socioeconomic status and facilitated access to healthcare and diagnostic modalities (21, 22). Also, genetic aberration is an important risk factor for thyroid cancer. For instance, a higher incidence of PIK3CA alterations was found in Middle Eastern papillary thyroid cancer patients (23). Moreover, increasing number of skilled endocrinologists, radiologists, and nuclear medicine specialists could be a potential factor for increasing the diagnosis and therefore the incidence of thyroid cancer.

We found a positive relationship between SDI and incidence of thyroid cancer. Of note, the increased capacity to diagnose during the study period might also be a reason for it, which needs to be evaluated in further studies. Some risk factors of thyroid cancer like high BMI, smoking, ambient air pollution, availability of diagnostic and screening modalities, and better access to healthcare services have been shown to have a positive relationship with developing the level of SDI, so it might explain the higher incidence of thyroid cancer in high SDI countries (4, 24).

Furthermore, we found that incidence and deaths due to thyroid cancer are much more common in women and they are positively correlated with age. Different global-, regional-, and national-scale studies as well as our study showed a higher incidence rate of thyroid cancer in women than men (25, 26). The specific mechanism for gender disparity of thyroid cancer has not been expressed clearly, whereas imbalance between two isoforms of estrogen receptors and the role of sex hormones were suggested (27). The results of the Global Cancer Incidence, Mortality, and Prevalence (GLOBOCAN) 2020 study showed that thyroid cancer in Turkey and Iran with 13,682 and 4,114 incident numbers among women had the highest values in the Eastern Mediterranean Region (EMR) (28), which is in accordance with our finding that these two countries had the highest incident numbers in women.

A large-scale observational study on patients with papillary thyroid cancer demonstrated that there is a continuous and linear fashion in mortality of thyroid cancer that certify our findings (29). The results of the GBD 2015 study in EMR also revealed that Afghanistan had the highest age-standardized death and DALY rate for all cancers in 2015 which was in accordance with our findings in 2019 about thyroid cancer (25). Poor socioeconomic status, unstable political system, and low-quality healthcare services might be some reasons to explain the higher deaths and DALYs of thyroid cancer in Afghanistan (30). On the other hand, Syrian Arab Republic, which encounters conflicts and wars, showed low incidence, death, and DALY rate both in 2015 (25) and in 2019, which needs further evaluation in order to determine whether it is because of underdiagnoses and lower quality of cancer registry systems or it is as a result of heath policies and programs (31). Also, the low incidence in Syria in 2019 might be due to an unstable political system and weak healthcare system in this war-affected area resulting in less availability of diagnostic facilities.

We included high BMI as a risk factor of thyroid cancer and estimated the burden of thyroid cancer attributable to it. It showed that about one-sixth percent of deaths and DALYs of thyroid cancer are attributable to high BMI. A pooled meta-analysis of 22 prospective studies including more than 2 million participants showed that greater levels of baseline BMI are associated with increased risk of thyroid cancer mortality (hazard ratio (HR) = 1.29; 95% confidence interval (CI): 1.07–1.55), which is in accordance with our study (32). Also, the population-based study by Arnold et al. showed that a rise at 5 kg/m^2^ in BMI increased the risk of developing thyroid cancer 1.33 and 1.14 in men and women, respectively (33). However, it is not the only risk factor for thyroid cancer incidence and deaths and there are others like radiation exposure, smoking, alcohol consumption, nutrition elements such as iodine, seafood, nitrate, and coffee, and history of underlying thyroid diseases that might contribute to thyroid cancer and attributable deaths (34). The global percent change of age-standardized death and DALY rate of thyroid cancer attributable to high BMI over 2006–2016 was estimated at 4.67 and 7.53, respectively, while we found a higher rate in the NAME region over 2010–2019 (percent change of age-standardized rate of death and DALY was 13.61 and 13.47, respectively) (24). It might be due to the higher prevalence of overweight and obesity in this region. The overall prevalence of obesity was 5% among children and 12% among adults in 2015 worldwide, whereas it was 4.9% and 20.7% among children and adults in the same year in EMR (35, 36). Also, the lowest level of physical activity, nutritional transition to Western foods, and higher levels of ambient air pollution in NAME are some risks for higher prevalence of excess body weight in this region (37).

To our knowledge, this is the most recent study describing regional burden of thyroid cancer and its attributable risk factors in the NAME region. However, our study has some limitations, which are related to methods used in GBD studies that were unavoidable. First, we did not describe the burden of thyroid cancer by each pathologic type such as papillary thyroid carcinoma, follicular thyroid carcinoma, medullary thyroid carcinoma, and anaplastic (undifferentiated) thyroid carcinoma or by their primary or secondary origin (38). Second, only the effects of BMI as a risk factor for thyroid cancers were evaluated, while other potential risk factors like radiation, iodine intake, autoimmune thyroiditis, thyroid nodules, insulin resistance, and other diet, lifestyles, and environmental pollutants were not included (39). Third, data sources in some countries might be scarce which could affect our regional estimations. Fourth, it seems that there is a possibility of information bias due to low diagnostic facilities and equipment for thyroid cancer. Considering the present limitations of GBD, this is the most accurate epidemiologic data we can access and utilize to study this region.

## Conclusions

Investigating the regional burden of thyroid cancer and its attributable risk factors is necessary for health policymakers because of cultural similarities and communications. Also, women, the elderly above about 60 years old, in the NAME region, and countries with a higher SDI showed a higher incidence rate. Considering the findings of this study, we recommend regional policymakers to provide high-level quality of care in high-prevalence thyroid cancer regions. Further studies are needed to determine more modifiable risk factors like potential harmful exposures attributable to mortality and incidence of thyroid cancer and to provide achievable and promising plans for thyroid cancer prevention. In future studies, a possible link of BMI with respect to genetic abnormalities and consequent cancer onsets in all countries across the globe should also be explored. Also, given the potential relationship between COVID-19 and cancer, it is advised that future GBD analyses explore the impact of COVID-19 pandemic on thyroid cancer burden.

## Data availability statement

The datasets presented in this study can be found in online repositories. The names of the repository/repositories and accession number(s) can be found below: https://vizhub.healthdata.org/gbd-results/.

## Ethics statement

The present report was approved by the ethics committee of the Endocrinology and Metabolism Research Institute, Tehran University of Medical Sciences, Tehran, Iran (IR.TUMS.EMRI.REC.1400.030). Written informed consent for participation was not required for this study in accordance with the national legislation and the institutional requirements.

## GBD 2019 NAME Thyroid Cancer Collaborators

**Seyed Aria Nejadghaderi**, Non-Communicable Diseases Research Center, Endocrinology and Metabolism Population Sciences Institute, Tehran University of Medical Sciences, Tehran, Iran; **Sahar Saeedi Moghaddam**, Non-Communicable Diseases Research Center, Endocrinology and Metabolism Population Sciences Institute, Tehran University of Medical Sciences, Tehran, Iran; **Sina Azadnajafabad**, Non-Communicable Diseases Research Center, Endocrinology and Metabolism Population Sciences Institute, Tehran University of Medical Sciences, Tehran, Iran; **Negar Rezaei**, Non-Communicable Diseases Research Center, Endocrinology and Metabolism Population Sciences Institute, Tehran University of Medical Sciences, Tehran, Iran; Endocrinology and Metabolism Research Center, Endocrinology and Metabolism Clinical Sciences Institute, Tehran University of Medical Sciences, Tehran, Iran; **Nazila Rezaei**, Non-Communicable Diseases Research Center, Endocrinology and Metabolism Population Sciences Institute, Tehran University of Medical Sciences, Tehran, Iran; **Mohsen Abbasi-Kangevari**, Non-Communicable Diseases Research Center, Endocrinology and Metabolism Population Sciences Institute, Tehran University of Medical Sciences, Tehran, Iran; **Zeinab Abbasi-Kangevari**, Non-Communicable Diseases Research Center, Endocrinology and Metabolism Population Sciences Institute, Tehran University of Medical Sciences, Tehran, Iran; Social Determinants of Health Research Center, Shahid Beheshti University of Medical Sciences, Tehran, Iran; **Sina Abdollahzade**, Department of Surgery, Qazvin University of Medical Sciences, Qazvin, Iran; Rajayi Hospital, Qazvin University of Medical Sciences, Qazvin, Iran; **Eman Abu-Gharbieh**, Clinical Sciences Department, University of Sharjah, Sharjah, United Arab Emirates; **Sima Afrashteh**, Department of Public Health, Bushehr University of Medical Sciences, Bushehr, Iran; Department of Epidemiology, Shiraz University of Medical Sciences, Shiraz, Iran; **Muhammad Sohail Afzal**, Department of Life Sciences, University of Management and Technology, Lahore, Pakistan; **Sajjad Ahmad**, Department of Health and Biological Sciences, Abasyn University, Peshawar, Pakistan; **Ali Ahmadi**, Department of Epidemiology and Biostatistics, Shahrekord University of Medical Sciences, Shahrekord, Iran; Department of Epidemiology, Shahid Beheshti University of Medical Sciences, Tehran, Iran; **Sepideh Ahmadi**, School of Advanced Technologies in Medicine, Shahid Beheshti University of Medical Sciences, Tehran, Iran; **Haroon Ahmed**, Department of Biosciences, COMSATS Institute of Information Technology, Islamabad, Pakistan; **Luai A Ahmed**, Institute of Public Health, United Arab Emirates University, Al Ain, United Arab Emirates; **Hanadi Al Hamad**, Geriatric and Long Term Care Department, Hamad Medical Corporation, Doha, Qatar; Rumailah Hospital, Hamad Medical Corporation, Doha, Qatar; **Fadwa Alhalaiqa Naji Alhalaiqa**, Faculty of Nursing, Philadelphia University, Amman, Jordan; Psychological Sciences Association, Amman, Jordan; **Saba Alvand**, Liver and Pancreatobiliary Diseases Research Center, Tehran University of Medical Sciences, Tehran, Iran; **Fazel Isapanah Amlashi**, Neuroscience Research Center, Golestan University of Medical Sciences, Gorgan, Iran; **Ali Arash Anoushirvani**, Department of Internal Medicine, Iran University of Medical Sciences, Tehran, Iran; **Jalal Arabloo**, Health Management and Economics Research Center, Iran University of Medical Sciences, Tehran, Iran; **Seyyed Shamsadin Athari**, Department of Immunology, Zanjan University of Medical Sciences, Zanjan, Iran; **Mohammadreza Azangou-Khyavy**, Social Determinants of Health Research Center, Shahid Beheshti University of Medical Sciences, Tehran, Iran; Non-Communicable Diseases Research Center, Endocrinology and Metabolism Population Sciences Institute, Tehran University of Medical Sciences, Tehran, Iran; **Amirhossein Azari Jafari**, School of Medicine, Shahroud University of Medical Sciences, Shahroud, Iran; **Ali Bijani**, Social Determinants of Health Research Center, Babol University of Medical Sciences, Babol, Iran; **Iman El Sayed**, Biomedical Informatics and Medical Statistics Department, Alexandria University, Alexandria, Egypt; **Iffat Elbarazi**Institute of Public Health, United Arab Emirates University, Al Ain, United Arab Emirates; **Muhammed Elhadi**, Faculty of Medicine, University of Tripoli, Tripoli, Libya; **Pawan Sirwan Faris**, Department of Biology, Salahaddin University-Erbil, Erbil, Iraq; Department of Biology, Cihan University-Erbil, Erbil, Iraq; **Abbas Farmany**, Department of Health Sciences, Hamadan University of Medical Sciences, Hamadan, Iran; **Ali Fatehizadeh**, Department of Environmental Health Engineering, Isfahan University of Medical Sciences, Isfahan, Iran; **Azin Ghamari**, Endocrinology and Metabolism Research Center, Endocrinology and Metabolism Clinical Sciences Institute, Tehran University of Medical Sciences, Tehran, Iran; **Seyyed-Hadi Ghamari**, Non-Communicable Diseases Research Center, Endocrinology and Metabolism Population Sciences Institute, Tehran University of Medical Sciences, Tehran, Iran; Social Determinants of Health Research Center, Shahid Beheshti University of Medical Sciences, Tehran, Iran; **Ahmad Ghashghaee**, School of Public Health, Qazvin University of Medical Sciences, Qazvin, Iran; **Pouya Goleij**, Department of Genetics, Sana Institute of Higher Education, Sari, Iran; **Mohamad Golitaleb**, Department of Nursing, Arak University of Medical Sciences, Arak, Iran; **Arvin Haj-Mirzaian**, Department of Pharmacology, Shahid Beheshti University of Medical Sciences, Tehran, Iran; Obesity Research Center, Shahid Beheshti University of Medical Sciences, Tehran, Iran; **Rabih Halwani**, Clinical Sciences Department, University of Sharjah, Sharjah, United Arab Emirates; College of Medicine, University of Sharjah, Sharjah, United Arab Emirates, **Samer Hamidi**, School of Health and Environmental Studies, Hamdan Bin Mohammed Smart University, Dubai, United Arab Emirates; **Soheil Hassanipour**, Gastrointestinal and Liver Diseases Research Center, Guilan University of Medical Sciences, Rasht, Iran; Caspian Digestive Disease Research Center, Guilan University of Medical Sciences, Rasht, Iran; **Mowafa Househ**, College of Science and Engineering, Hamad Bin Khalifa University, Doha, Qatar; **Tahereh Javaheri**, Health Informatics Lab, Boston University, Boston, MA, USA; **Taras Kavetskyy**, Department of Surface Engineering, The John Paul II Catholic University of Lublin, Lublin, Poland; Drohobych Ivan Franko State Pedagogical University, Drohobych, Ukraine; **Rovshan Khalilov**, Department of Biophysics and Biochemistry, Baku State University, Baku, Azerbaijan; Russian Institute for Advanced Study, Moscow State Pedagogical University, Moscow, Russia; **Ejaz Ahmad Khan**, Department of Epidemiology and Biostatistics, Health Services Academy, Islamabad, Pakistan; **Javad Khanali**, Social Determinants of Health Research Center, Shahid Beheshti University of Medical Sciences, Tehran, Iran; Non-Communicable Diseases Research Center, Endocrinology and Metabolism Population Sciences Institute, Tehran University of Medical Sciences, Tehran, Iran; **Maryam Khayamzadeh**, Shahid Beheshti University of Medical Sciences, Tehran, Iran; The Iranian Academy of Medical Sciences, Tehran, Iran; **Ali-Asghar Kolahi**, Social Determinants of Health Research Center, Shahid Beheshti University of Medical Sciences, Tehran, Iran; Department of Health & Community Medicine, Shahid Beheshti University of Medical Sciences, Tehran, Iran; **Hamid Reza Koohestani**, Social Determinants of Health Research Center, Saveh University of Medical Sciences, Saveh, Iran; **Somayeh Livani**, Radiology Department, Golestan University of Medical Sciences, Gorgan, Iran; **Mohammad-Reza Malekpour**, Non-Communicable Diseases Research Center, Endocrinology and Metabolism Population Sciences Institute, Tehran University of Medical Sciences, Tehran, Iran; **Ahmad Azam Malik**, Rabigh Faculty of Medicine, King Abdulaziz University, Jeddah, Saudi Arabia; University Institute of Public Health, The University of Lahore, Lahore, Pakistan; **Entezar Mehrabi Nasab**, Tehran Heart Center, Tehran University of Medical Sciences, Tehran, Iran; **Seyyedmohammadsadeq Mirmoeeni**, School of Medicine, Shahroud University of Medical Sciences, Shahroud, Iran; **Yousef Mohammad**, Internal Medicine Department, King Saud University, Riyadh, Saudi Arabia; **Esmaeil Mohammadi**, Non-Communicable Diseases Research Center, Endocrinology and Metabolism Population Sciences Institute, Tehran University of Medical Sciences, Tehran, Iran; Faculty of Medicine, Tehran University of Medical Sciences, Tehran, Iran; **Abdollah Mohammadian-Hafshejani**, Department of Epidemiology and Biostatistics, Shahrekord University of Medical Sciences, Shahrekord, Iran; **Sara Momtazmanesh**, School of Medicine, Tehran University of Medical Sciences, Tehran, Iran; Non-Communicable Diseases Research Center, Endocrinology and Metabolism Population Sciences Institute, Tehran University of Medical Sciences, Tehran, Iran; **Paula Moraga**, Computer, Electrical, and Mathematical Sciences and Engineering Division, King Abdullah University of Science and Technology, Thuwal, Saudi Arabia; **Zuhair S Natto**, Department of Dental Public Health, King Abdulaziz University, Jeddah, Saudi Arabia; Department of Health Policy and Oral Epidemiology, Harvard University, Boston, MA, USA; **Maryam Noori**, Student Research Committee, Iran University of Medical Sciences, Tehran, Iran; **Ali Nowroozi**, School of Medicine, Tehran University of Medical Sciences, Tehran, Iran; **Fatemeh Pashazadeh Kan**, Iran University of Medical Sciences, Tehran, Iran; **Zahra Zahid Piracha**, Department of Public Health, Health Services Academy, Islamabad, Pakistan; **Sima Rafiei**, Social Determinants of Health Research Center, Qazvin University of Medical Sciences, Qazvin, Iran; **Kiana Ramezanzadeh**, Department of Pharmacology, Shahid Beheshti University of Medical Sciences, Tehran, Iran; **Mahsa Rashidi**, Department of Clinical Science, Islamic Azad University, Garmsar, Iran; **Mohammad-Mahdi Rashidi**, Non-Communicable Diseases Research Center, Endocrinology and Metabolism Population Sciences Institute, Tehran University of Medical Sciences, Tehran, Iran; Social Determinants of Health Research Center, Shahid Beheshti University of Medical Sciences, Tehran, Iran; **Reza Rawassizadeh**, Department of Computer Science, Boston University, Boston, MA, USA; **Nima Rezaei**, Research Center for Immunodeficiencies, Tehran University of Medical Sciences, Tehran, Iran; Network of Immunity in Infection, Malignancy and Autoimmunity (NIIMA), Universal Scientific Education and Research Network (USERN), Tehran, Iran; **Sahba Rezazadeh-Khadem**, Non-Communicable Diseases Research Center, Endocrinology and Metabolism Population Sciences Institute, Tehran University of Medical Sciences, Tehran, Iran; **Basema Saddik**, Sharjah Institute for Medical Research, University of Sharjah, Sharjah, United Arab Emirates; **Umar Saeed**, Research and Development, Islamabad Diagnostic Center Pakistan, Islamabad, Pakistan; Biological Production Division, National Institute of Health, Islamabad, Pakistan; **Amirhossein Sahebkar**, Applied Biomedical Research Center, Mashhad University of Medical Sciences, Mashhad, Iran; Biotechnology Research Center, Mashhad University of Medical Sciences, Mashhad, Iran; **Abdallah M Samy**, Department of Entomology, Ain Shams University, Cairo, Egypt; **Muhammad Arif Nadeem Saqib**, Research Development Coordination Section, Pakistan Health Research Council, Islamabad, Pakistan; School of Sciences, University of Management and Technology, Lahore, Pakistan; **Brijesh Sathian**, Geriatric and Long Term Care Department, Hamad Medical Corporation, Doha, Qatar; Faculty of Health & Social Sciences, Bournemouth University, Bournemouth, UK; **Saeed Shahabi**, Health Policy Research Center, Shiraz University of Medical Sciences, Shiraz, Iran; **Sarvenaz Shahin**, Non-Communicable Diseases Research Center, Endocrinology and Metabolism Population Sciences Institute, Tehran University of Medical Sciences, Tehran, Iran; **Elaheh Shaker**, Non-Communicable Diseases Research Center, Endocrinology and Metabolism Population Sciences Institute, Tehran University of Medical Sciences, Tehran, Iran; School of Medicine, Tehran University of Medical Sciences, Tehran, Iran; **Javad Sharifi-Rad**, Phytochemistry Research Center, Shahid Beheshti University of Medical Sciences, Tehran, Iran; **Parnian Shobeiri**, Non-Communicable Diseases Research Center, Endocrinology and Metabolism Population Sciences Institute, Tehran University of Medical Sciences, Tehran, Iran; School of Medicine, Tehran University of Medical Sciences, Tehran, Iran; **Yasaman TaheriAbkenar**, Phytochemistry Research Center, Shahid Beheshti University of Medical Sciences, Tehran, Iran; **Iman M Talaat**, Clinical Sciences Department, University of Sharjah, Sharjah, United Arab Emirates; Pathology Department, Alexandria University, Alexandria, Egypt; **Irfan Ullah**, Department of Life Sciences, University of Management and Technology, Lahore, Pakistan; Pakistan Council for Science and Technology, Ministry of Science and Technology, Islamabad, Pakistan; **Rohollah Valizadeh**, Department of Epidemiology, Iran University of Medical Sciences, Tehran, Iran, **Bay Vo**, Faculty of Information Technology, HUTECH University, Ho Chi Minh City, Vietnam; **Deniz Yuce**, Cancer Institute, Hacettepe University, Ankara, Turkey; **Iman Zare**, Research and Development Department, Sina Medical Biochemistry Technologies, Shiraz, Iran; **Seyed Mohammad Tavangar**, Department of Pathology, Tehran University of Medical Sciences, Tehran, Iran; **Hamidreza Jamshidi**, Department of Pharmacology, Shahid Beheshti University of Medical Sciences, Tehran, Iran; Ministry of Health and Medical Education, Tehran, Iran; **Ali H Mokdad**, Institute for Health Metrics and Evaluation, University of Washington, Seattle, WA, USA; Department of Health Metrics Sciences, School of Medicine, University of Washington, Seattle, WA, USA; **Mohsen Naghavi**, Institute for Health Metrics and Evaluation, University of Washington, Seattle, WA, USA; Department of Health Metrics Sciences, School of Medicine, University of Washington, Seattle, WA, USA; **Farshad Farzadfar**, Non-Communicable Diseases Research Center, Endocrinology and Metabolism Population Sciences Institute, Tehran University of Medical Sciences, Tehran, Iran; Endocrinology and Metabolism Research Center, Endocrinology and Metabolism Clinical Sciences Institute, Tehran University of Medical Sciences, Tehran, Iran; **Bagher Larijani**, Endocrinology and Metabolism Research Center, Endocrinology and Metabolism Clinical Sciences Institute, Tehran University of Medical Sciences, Tehran, Iran.

## Author contributions

All authors listed have made a substantial, direct, and intellectual contribution to the work and approved it for publication.

## Funding

The Bill and Melinda Gates Foundation, who were not involved in any way in the preparation of this manuscript, funded the GBD study.

## Acknowledgments

We would like to thank the Institute for Health Metrics and Evaluation staff and its collaborators who prepared these publicly available data. Z Piracha and U Saeed would like to acknowledge the support of the International Center of Medical Sciences Research, Islamabad (44000), Pakistan. A M Samy acknowledges the support from the Egyptian Fulbright Mission Program and being a member of the Egyptian Young Academy of Sciences.

## Conflict of interest

The authors declare that the research was conducted in the absence of any commercial or financial relationships that could be construed as a potential conflict of interest.

## Publisher’s note

All claims expressed in this article are solely those of the authors and do not necessarily represent those of their affiliated organizations, or those of the publisher, the editors and the reviewers. Any product that may be evaluated in this article, or claim that may be made by its manufacturer, is not guaranteed or endorsed by the publisher.

## Author disclaimer

This study is based on publicly available data and solely reflects the opinion of its authors and not that of the Institute for Health Metrics and Evaluation.

## Supplementary material

Supplementary Figure 1 Age-standardized rate of incidence, prevalence, deaths, and disability-adjusted life years (DALYs) of thyroid cancer in North Africa and Middle East region among both sexes in 1990, by country.

Supplementary Figure 2 Age-standardized rate of incidence, prevalence, deaths, and disability-adjusted life years (DALYs) of thyroid cancer in North Africa and Middle East region among both sexes in 2019, by country.

Supplementary Figure 3 Trend of age-standardized rate of incidence, prevalence, deaths, and disability-adjusted life years (DALYs) of thyroid cancer in North Africa and Middle East region among both sexes over 1990-2019, by country.

Supplementary Figure 4 Age-standardized percent of disability-adjusted life years (DALYs) of thyroid cancer attributable to high body mass index (BMI) in North Africa and Middle East region among both sexes in 1990 and 2019, by country.

Supplementary Figure 5 Age-standardized percent of deaths of thyroid cancer attributable to high body mass index (BMI) in North Africa and Middle East region among both sexes in 1990 and 2019, by country.

Supplementary Figure 6 Age-standardized rate of incidence of thyroid cancer in North Africa and Middle East region among both sexes in 1990 and 2019, by health access and quality index (HAQI).

Supplementary Figure 7 Age-standardized rate of death of thyroid cancer in North Africa and Middle East region among both sexes in 1990 and 2019, by health access and quality index (HAQI).

Supplementary Figure 8 Age-standardized rate of disability-adjusted life years (DALYs) of thyroid cancer in North Africa and Middle East region among both sexes in 1990 and 2019, by health access and quality index (HAQI).
