# Erratum: Burden of thyroid cancer in North Africa and Middle East 1990–2019

**DOI:** 10.3389/fonc.2023.1208646

**Published:** 2023-04-28

**Authors:** Frontiers Production Office

**Affiliations:** Frontiers Media SA, Lausanne, Switzerland

**Keywords:** north africa and middle east, thyroid cancer, incidence, mortality, disability-adjusted life years, body mass index, risk factor

Due to a production error, there was mistake in the author list. In the published article, the GBD 2019 NAME Thyroid Cancer Collaborators should have been credited with authorship. Thus, in the author list, “on behalf of GBD 2019 NAME Thyroid Cancer Collaborators” has now been corrected to “and GBD 2019 NAME Thyroid Cancer Collaborators”.

The publisher apologizes for this mistake. The original version of this article has been updated.

