# Supplementary material for: Burden of thyroid cancer in North Africa and Middle East 1990–2019
Source: Front Oncol. 2022 Sep 23;12:955358. doi: 10.3389/fonc.2022.955358 (PMC9538696; doi:10.3389/fonc.2022.955358)
Supplement: Supplementary Figure 1 — Age-standardized rate of incidence, prevalence, deaths, and disability-adjusted life years (DALYs) of thyroid cancer in North Africa and Middle East region among both sexes in 1990, by country. [file DataSheet_1.pdf]

Providing data or critical feedback on data sources

Sina Abdollahzade, Muhammad Sohail Afzal, Sajjad Ahmad, Ali Ahmadi, Sepideh Ahmadi, Hanadi Al Hamad, Fadwa Alhalaiqa Naji Alhalaiqa, Fazel Isapanah Amlashi, Jalal Arabloo, Seyyed Shamsadin Athari, Iman El Sayed, Farshad Farzadfar, Ahmad Ghashghaee, Pouya Goleij, Arvin Haj-Mirzaian, Soheil Hassanipour, Mowafa Househ, Tahereh Javaheri, Taras Kavetskyy, Rovshan Khalilov, Ali-Asghar Kolahi, Somayeh Livani, Entezar Mehrabi Nasab, Abdollah Mohammadian-Hafshejani, Ali H Mokdad, Mohsen Naghavi, Zuhair S Natto, Maryam Noori, Fatemeh Pashazadeh Kan, Zahra Zahid Piracha, Sima Rafiei, Kiana Ramezanzadeh, Reza Rawassizadeh, Nima Rezaei, Basema Saddik, Umar Saeed, Sahar Saeedi Moghaddam, Abdallah M Samy, Brijesh Sathian, Sarvenaz Shahin, Javad Sharifi-Rad, Parnian Shobeiri, Yasaman TaheriAbkenar, Irfan Ullah, Bay Vo, Deniz Yuce, and Iman Zare.

Developing methods or computational machinery

Ali H Mokdad, Mohsen Naghavi, and Sahar Saeedi Moghaddam.

Providing critical feedback on methods or results

Muhammad Sohail Afzal, Sajjad Ahmad, Ali Ahmadi, Haroon Ahmed, Luai A Ahmed, Hanadi Al Hamad, Fadwa Alhalaiqa Naji Alhalaiqa, Saba Alvand, Fazel Isapanah Amlashi, Ali Arash Anoushirvani, Jalal Arabloo, Seyyed Shamsadin Athari, Sina Azadnajafabad, Mohammadreza Azangou-Khyavy, Amirhossein Azari Jafari, Ali Bijani, Iman El Sayed, Muhammed Elhadi, Pawan Sirwan Faris, Abbas Farmany, Farshad Farzadfar, Ali Fatehizadeh, Azin Ghamari, Ahmad Ghashghaee, Mohamad Golitaleb, Arvin Haj-Mirzaian, Rabih Halwani, Samer Hamidi, Soheil Hassanipour, Mowafa Househ, Hamidreza Jamshidi, Tahereh Javaheri, Taras Kavetskyy, Rovshan Khalilov, Ejaz Ahmad Khan, Javad Khanali, Maryam Khayamzadeh, Hamid Reza Koohestani, Bagher Larijani, Somayeh Livani, Mohammad-Reza Malekpour, Ahmad Azam Malik, Entezar Mehrabi Nasab, Seyyedmohammadsadeq Mirmoeeni, Yousef Mohammad, Esmaeil Mohammadi, Abdollah Mohammadian-Hafshejani, Ali H Mokdad, Sara Momtazmanesh, Mohsen Naghavi, Zuhair S Natto, Seyed Aria Nejadghaderi, Ali Nowroozi, Fatemeh Pashazadeh Kan, Zahra Zahid Piracha, Kiana Ramezanzadeh, Mahsa Rashidi, Mohammad-Mahdi Rashidi, Reza Rawassizadeh, Negar Rezaei, Nima Rezaei, Sahba Rezazadeh-Khadem, Basema Saddik, Umar Saeed, Sahar Saeedi Moghaddam, Abdallah M Samy, Muhammad Arif Nadeem Saqib, Brijesh Sathian, Saeed Shahabi, Sarvenaz Shahin, Elaheh Shaker, Javad Sharifi-Rad, Parnian Shobeiri, Yasaman

TaheriAbkenar, Seyed Mohammad Tavangar, Irfan Ullah, Rohollah Valizadeh, Bay Vo, and Deniz Yuce.

Drafting the work or revising is critically for important intellectual content

Mohsen Abbasi-Kangevari, Zeinab Abbasi-Kangevari, Eman Abu-Gharbieh, Sima Afrashteh, Muhammad Sohail Afzal, Ali Ahmadi, Sepideh Ahmadi, Luai A Ahmed, Fadwa Alhalaiqa Naji Alhalaiqa, Saba Alvand, Fazel Isapanah Amlashi, Ali Arash Anoushirvani, Jalal Arabloo, Sina Azadnajafabad, Mohammadreza Azangou-Khyavy, Amirhossein Azari Jafari, Iman El Sayed, Iffat Elbarazi, Muhammed Elhadi, Farshad Farzadfar, Ali Fatehizadeh, Seyyed-Hadi Ghamari, Ahmad Ghashghaee, Mohamad Golitaleb, Arvin Haj-Mirzaian, Rabih Halwani, Mowafa Househ, Hamidreza Jamshidi, Taras Kavetskyy, Rovshan Khalilov, Ejaz Ahmad Khan, Javad Khanali, Bagher Larijani, Somayeh Livani, Mohammad-Reza Malekpour, Ahmad Azam Malik, Seyyedmohammadsadeq Mirmoeeni, Yousef Mohammad, Esmaeil Mohammadi, Abdollah Mohammadian-Hafshejani, Ali H Mokdad, Sara Momtazmanesh, Paula Moraga, Mohsen Naghavi, Zuhair S Natto, Seyed Aria Nejadghaderi, Ali Nowroozi, Fatemeh Pashazadeh Kan, Zahra Zahid Piracha, Kiana Ramezanzadeh, Nazila Rezaei, Negar Rezaei, Nima Rezaei, Sahba Rezazadeh-Khadem, Basema Saddik, Umar Saeed, Sahar Saeedi Moghaddam, Amirhossein Sahebkar, Abdallah M Samy, Saeed Shahabi, Elaheh Shaker, Javad Sharifi-Rad, Parnian Shobeiri, Yasaman TaheriAbkenar, Iman M Talaat, Seyed Mohammad Tavangar, Irfan Ullah, Rohollah Valizadeh, Deniz Yuce, and Iman Zare.

Managing the estimation or publications process

Sina Azadnajafabad, Farshad Farzadfar, Bagher Larijani, Ali H Mokdad, Mohsen Naghavi, Seyed Aria Nejadghaderi, Nazila Rezaei, Negar Rezaei, and Sahar Saeedi Moghaddam.

Supplementary Figure 1

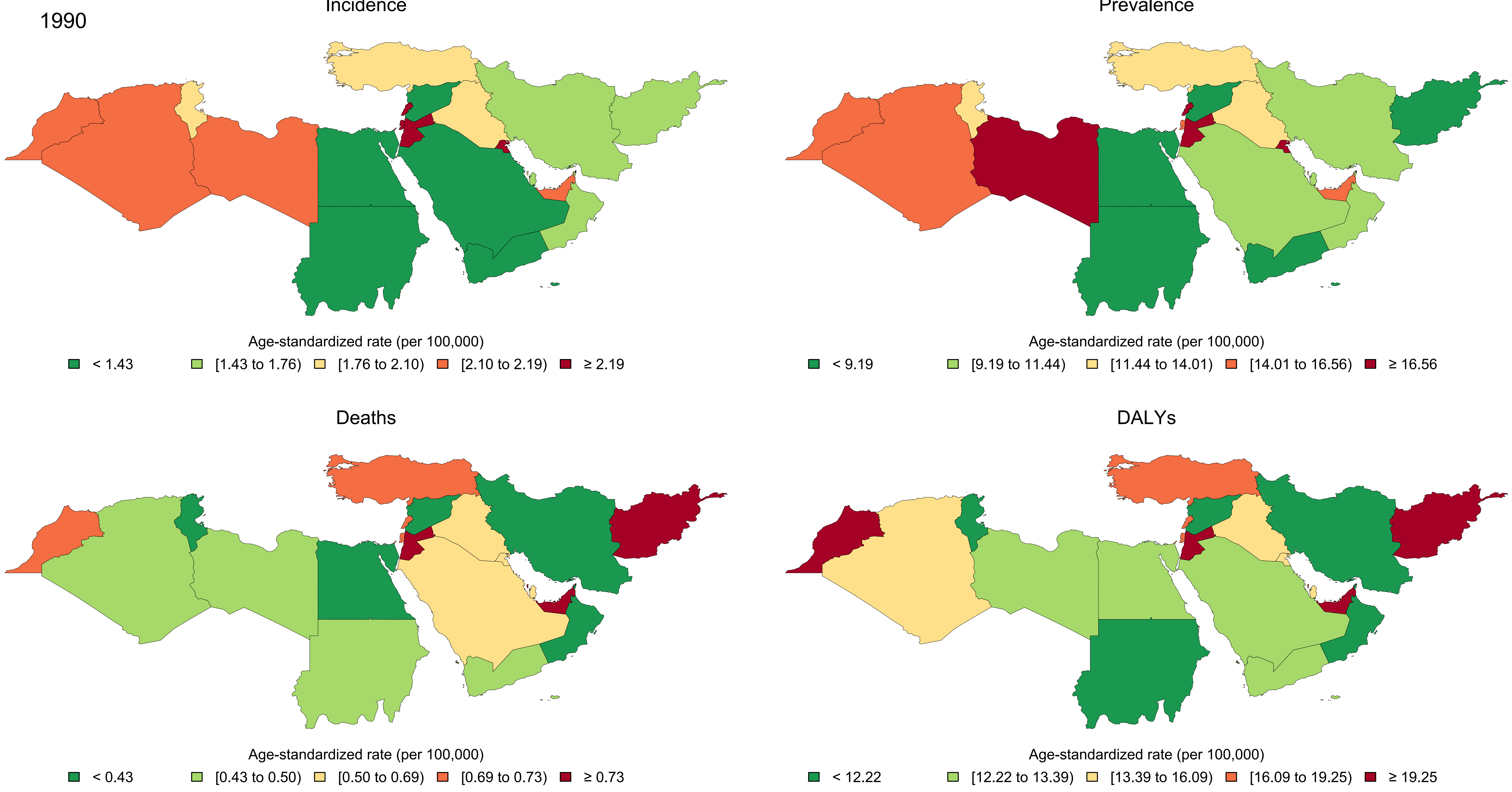

Supplementary Figure 2

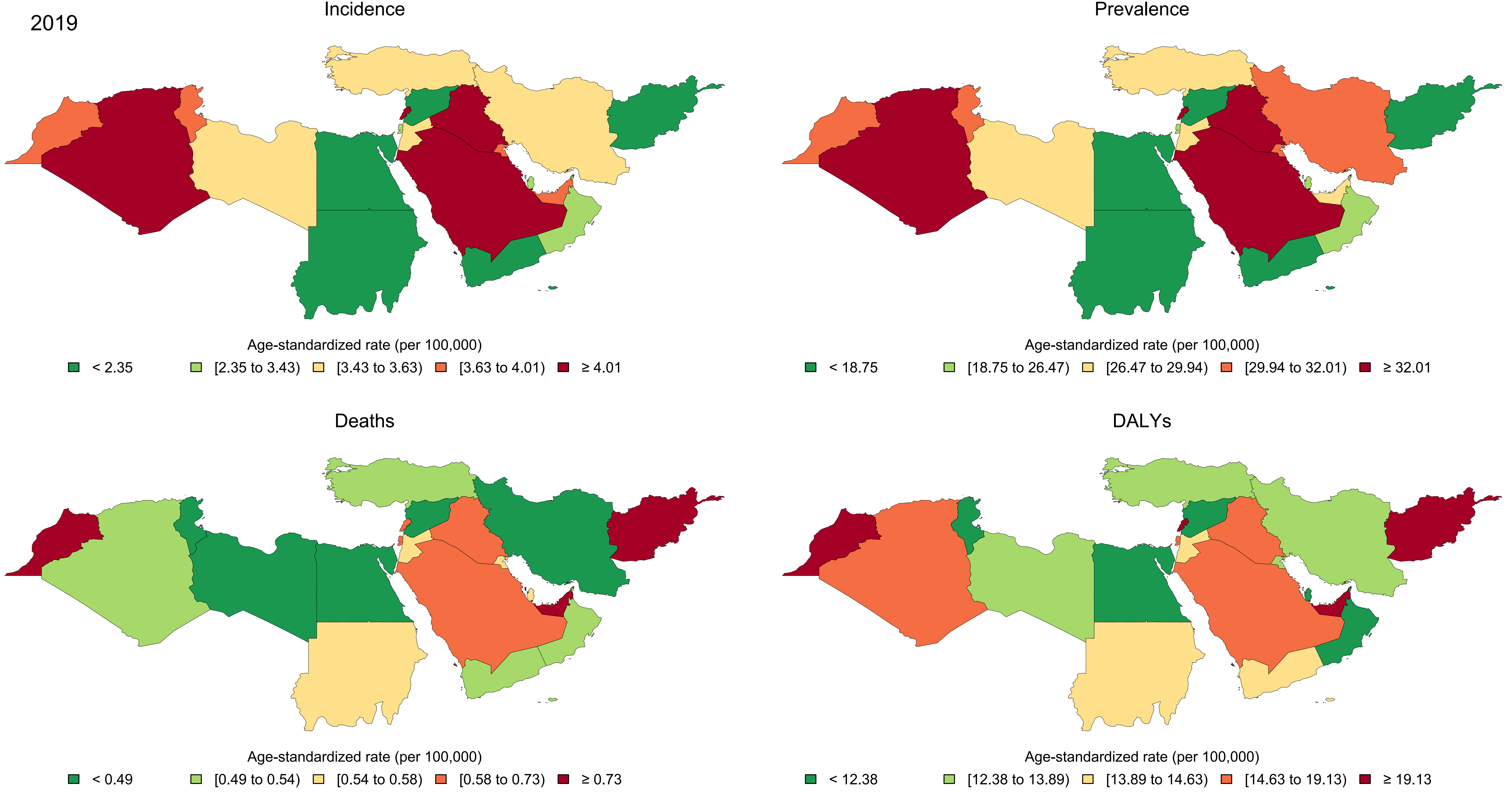

Supplementary Figure 3

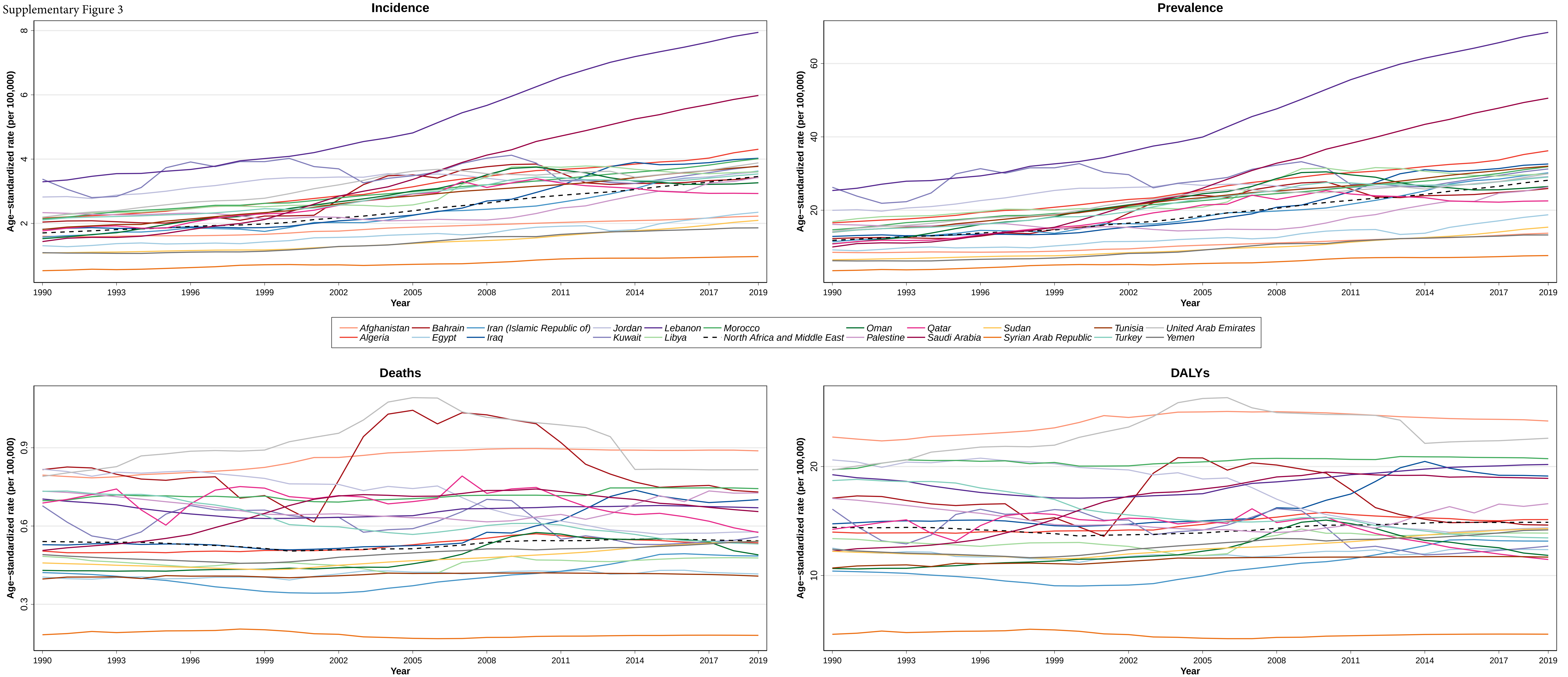

Supplementary Figure 4

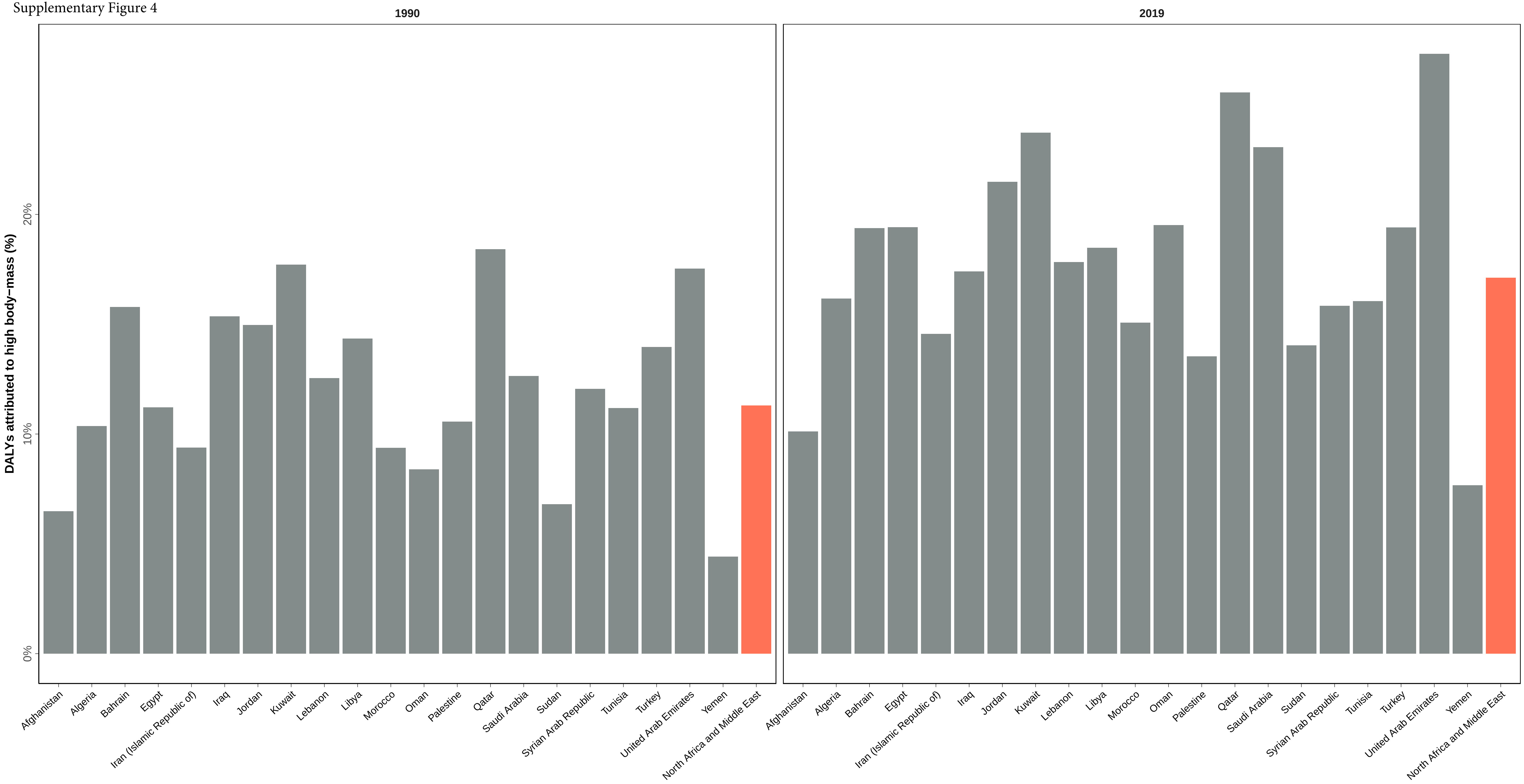

Supplementary Figure 5

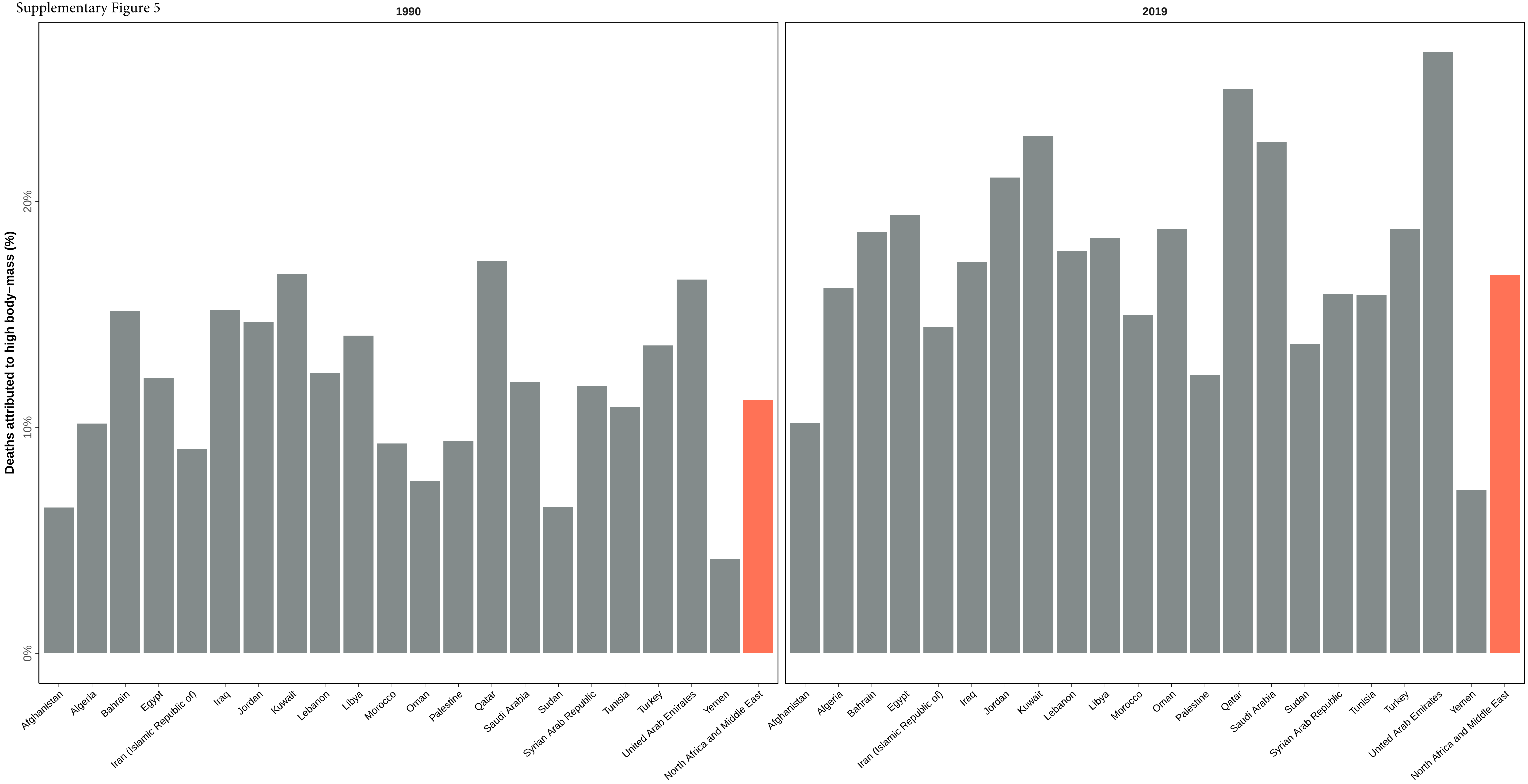

Supplementary Figure 6

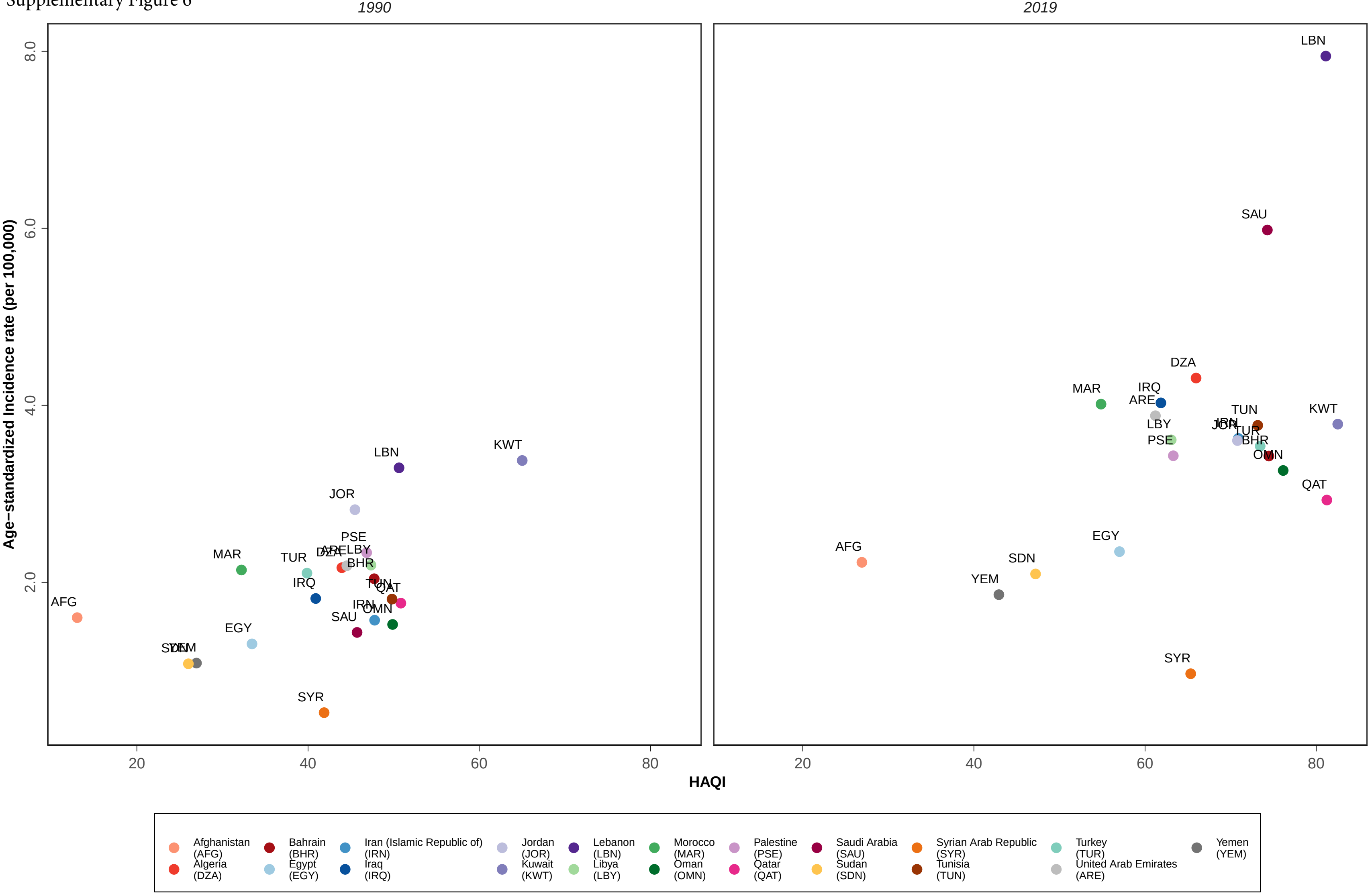

Supplementary Figure 7

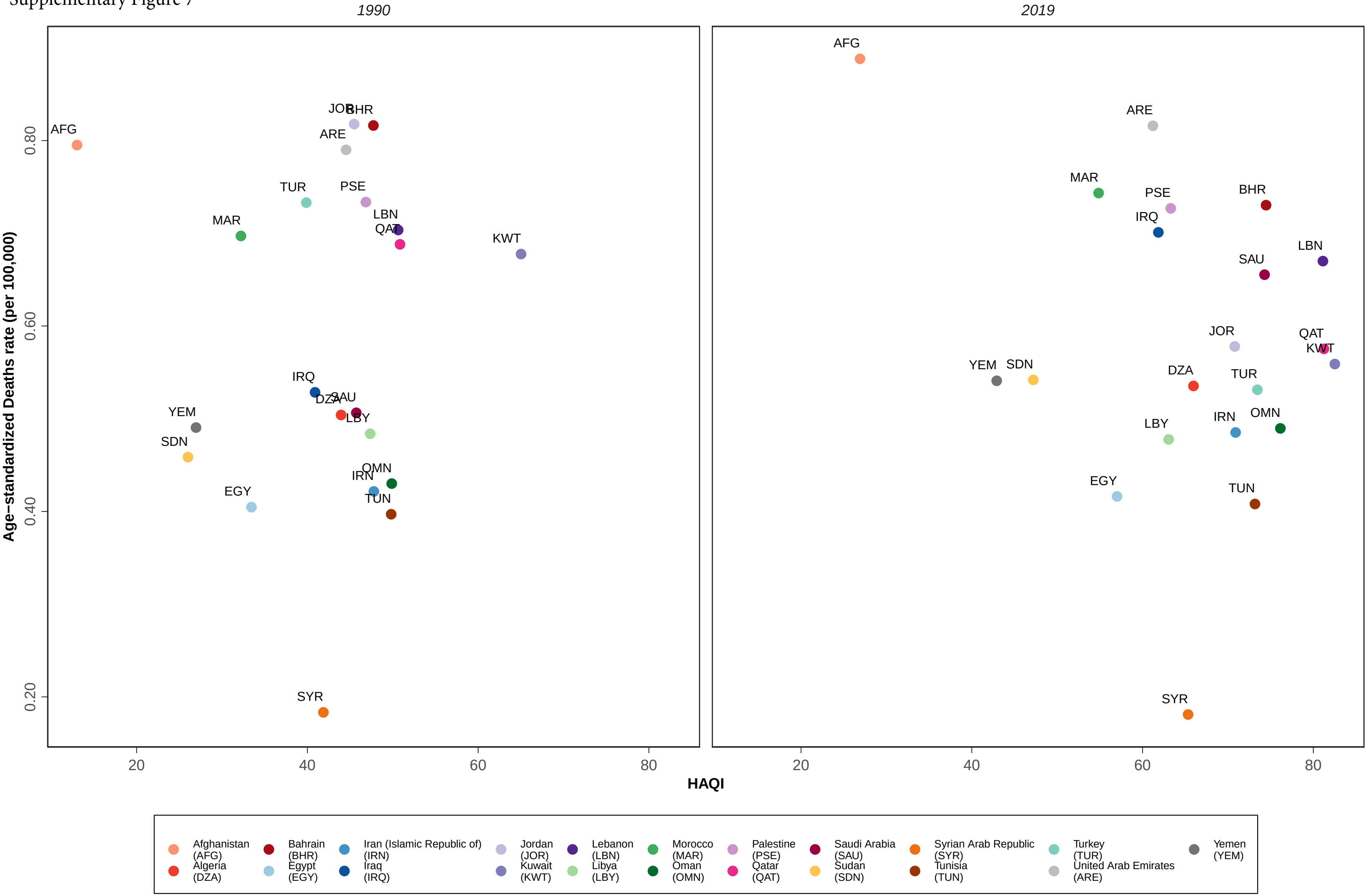

Supplementary Figure 8

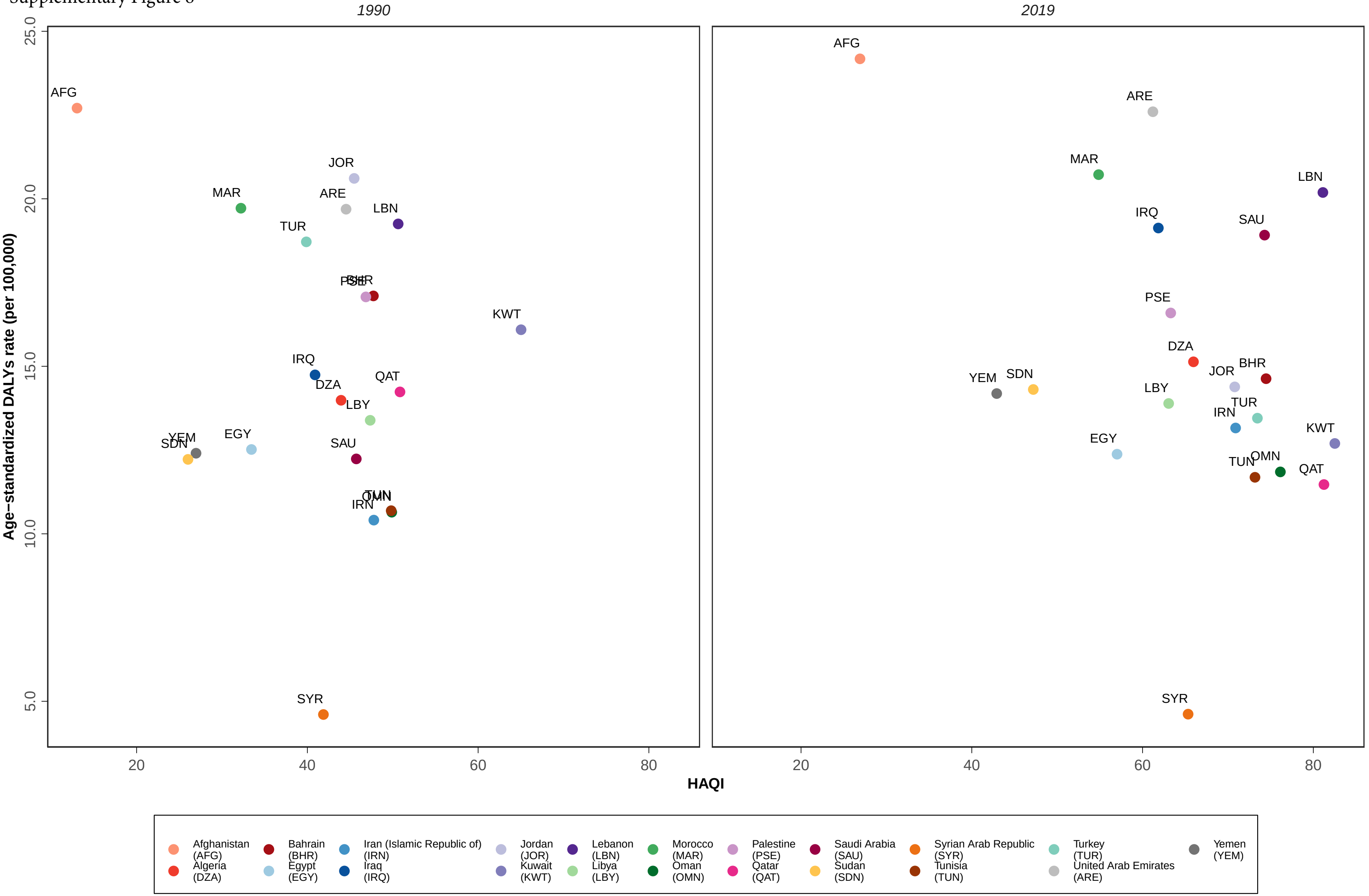

Table S1. Incidence, death, and disability-adjusted life years (DALYs) number and rate of thyroid cancer in North Africa and Middle East region in 1990 and 2019, by sex and country.

Location	Sex	1990						2019					
		Incidence		Deaths		DALYs (Disability-Adjusted Life Years)		Incidence		Deaths		DALYs (Disability-Adjusted Life Years)	
		Number	Rate	Number	Rate	Number	Rate	Number	Rate	Number	Rate	Number	Rate
North Africa and Middle East	Female	2970 (2361 to 3493)	2.6 (2.1 to 3.1)	608 (485 to 802)	0.7 (0.6 to 1)	19851 (16077 to 23713)	19.3 (15.4 to 24.4)	13586 (10441 to 15925)	5.1 (4 to 5.9)	1319 (1133 to 1589)	0.6 (0.6 to 0.8)	43117 (35094 to 50876)	17.9 (14.9 to 21)
	Male	911 (756 to 1172)	0.8 (0.7 to 1.1)	316 (255 to 425)	0.4 (0.3 to 0.5)	10473 (8581 to 13864)	9.7 (7.8 to 13.1)	5667 (4601 to 6669)	2 (1.6 to 2.3)	970 (820 to 1149)	0.4 (0.4 to 0.5)	31063 (25897 to 36269)	12 (10.1 to 14)
Afghanistan	Female	100 (43 to 168)	2.6 (1.1 to 4.2)	41 (21 to 65)	1.2 (0.6 to 1.9)	1323 (594 to 2189)	34 (15.8 to 55.6)	364 (154 to 635)	3.6 (1.7 to 5.9)	87 (46 to 137)	1.3 (0.8 to 1.9)	3119 (1471 to 5216)	34.9 (18.2 to 54.8)
	Male	22 (13 to 40)	0.6 (0.3 to 1)	15 (9 to 27)	0.4 (0.2 to 0.7)	446 (254 to 812)	11.2 (6.4 to 20.5)	75 (47 to 120)	0.8 (0.5 to 1.3)	31 (20 to 48)	0.5 (0.3 to 0.7)	1110 (706 to 1742)	12.9 (8.2 to 19.9)
Algeria	Female	312 (221 to 424)	3.6 (2.7 to 4.8)	42 (32 to 62)	0.7 (0.5 to 1.2)	1579 (1196 to 2083)	20 (15.4 to 27.7)	1484 (895 to 2172)	7 (4.4 to 9.9)	110 (83 to 145)	0.7 (0.6 to 0.9)	3940 (2684 to 5363)	20.7 (14.8 to 27.3)
	Male	62 (47 to 85)	0.8 (0.6 to 1)	18 (14 to 26)	0.3 (0.2 to 0.5)	617 (475 to 827)	8.1 (6.1 to 11.1)	343 (244 to 452)	1.7 (1.2 to 2.2)	62 (46 to 80)	0.4 (0.3 to 0.5)	1856 (1381 to 2432)	9.8 (7.3 to 12.7)
Bahrain	Female	4 (3 to 5)	3.5 (2.7 to 4.4)	1 (1 to 1)	1.1 (0.9 to 1.4)	21 (16 to 27)	24.6 (19 to 30.3)	32 (23 to 42)	6.5 (4.6 to 8.5)	3 (2 to 4)	1.1 (0.6 to 1.4)	85 (59 to 111)	23 (14.1 to 30.1)
	Male	1 (1 to 2)	0.9 (0.8	0 (0 to 1)	0.5 (0.4	12 (10 to 16)	10.7 (8.4	14 (9 to 19)	1.5 (1 to 1.9)	2 (1 to 2)	0.4 (0.3	57 (41 to 78)	8.4 (6.2

			to 1.2)		to 0.6)		to 13.6)				to 0.5)		to 11.1)
Egypt	Female	434 (330 to 561)	2 (1.6 to 2.5)	85 (70 to 127)	0.5 (0.4 to 0.9)	3358 (2658 to 4340)	16.4 (13.5 to 22.4)	1329 (891 to 1894)	3.3 (2.2 to 4.8)	143 (96 to 228)	0.5 (0.3 to 0.9)	5075 (3474 to 7345)	14.4 (9.8 to 22)
	Male	141 (111 to 181)	0.6 (0.5 to 0.9)	46 (38 to 68)	0.3 (0.2 to 0.5)	1878 (1517 to 2377)	8.7 (7.2 to 11.8)	679 (435 to 959)	1.5 (1 to 2.1)	128 (82 to 214)	0.3 (0.2 to 0.6)	4622 (2995 to 6591)	10.9 (7.1 to 16.4)
Iran (Islamic Republic of)	Female	400 (318 to 496)	2.4 (2 to 2.9)	62 (51 to 79)	0.5 (0.4 to 0.8)	1934 (1630 to 2311)	13.5 (11.3 to 17.1)	2242 (1310 to 2643)	5.1 (3.1 to 6)	195 (131 to 220)	0.6 (0.4 to 0.6)	5995 (3638 to 6924)	15.2 (9.5 to 17.4)
	Male	139 (110 to 183)	0.8 (0.6 to 1)	38 (30 to 50)	0.3 (0.2 to 0.4)	1239 (1013 to 1598)	7.4 (6 to 9.8)	956 (634 to 1129)	2.1 (1.5 to 2.5)	147 (112 to 163)	0.4 (0.3 to 0.5)	4474 (3132 to 5029)	11.1 (8 to 12.4)
Iraq	Female	138 (96 to 196)	2.6 (1.8 to 3.8)	25 (18 to 40)	0.6 (0.4 to 1)	844 (602 to 1209)	17.7 (12.6 to 26.5)	915 (597 to 1329)	5.7 (3.9 to 8)	85 (63 to 110)	0.7 (0.5 to 0.9)	2981 (2047 to 4097)	21.1 (15 to 28.1)
	Male	51 (36 to 71)	1 (0.7 to 1.4)	17 (13 to 24)	0.4 (0.3 to 0.6)	556 (407 to 783)	11.8 (8.6 to 16.6)	367 (252 to 510)	2.4 (1.7 to 3.2)	74 (54 to 93)	0.7 (0.5 to 0.8)	2309 (1638 to 3082)	17.2 (12.5 to 22.3)
Jordan	Female	46 (33 to 62)	4.6 (3.5 to 6.2)	7 (6 to 9)	1.1 (0.9 to 1.5)	243 (184 to 313)	29.1 (22.8 to 38.1)	229 (164 to 319)	5.6 (4 to 7.7)	21 (15 to 28)	0.8 (0.6 to 1)	640 (470 to 866)	18.5 (13.7 to 25)
	Male	11 (9 to 15)	1.1 (0.9 to 1.5)	3 (3 to 4)	0.5 (0.4 to 0.6)	112 (85 to 145)	12.4 (9.6 to 16.1)	93 (67 to 125)	1.9 (1.4 to 2.5)	14 (10 to 19)	0.4 (0.3 to 0.6)	444 (326 to 588)	10.6 (7.8 to 14.2)
Kuwait	Female	28 (23 to 33)	6.9 (5.8 to 8)	2 (2 to 3)	1.1 (0.9 to 1.3)	82 (70 to 95)	27.2 (22.9 to 31.6)	95 (71 to 129)	5.6 (4.4 to 7.3)	5 (4 to 7)	0.6 (0.5 to 0.8)	179 (139 to 236)	14.8 (11.7 to 19.1)

	Male	9 (7 to 10)	1.3 (1.1 to 1.5)	1 (1 to 2)	0.4 (0.3 to 0.4)	47 (40 to 54)	9.1 (7.7 to 10.5)	54 (41 to 73)	2.5 (1.9 to 3.2)	7 (5 to 8)	0.5 (0.4 to 0.6)	193 (146 to 249)	11.3 (8.6 to 14.4)
Lebanon	Female	67 (47 to 93)	5 (3.5 to 6.8)	10 (7 to 14)	0.9 (0.6 to 1.3)	310 (226 to 412)	24.4 (18 to 33.3)	308 (204 to 443)	10.7 (7.1 to 15.4)	18 (14 to 26)	0.6 (0.5 to 0.9)	603 (441 to 844)	21.2 (15.4 to 29.6)
	Male	19 (14 to 25)	1.5 (1.1 to 2)	6 (4 to 8)	0.5 (0.4 to 0.7)	170 (128 to 227)	13.8 (10.5 to 18.5)	123 (85 to 164)	4.9 (3.4 to 6.5)	17 (12 to 22)	0.7 (0.5 to 0.9)	467 (332 to 604)	19.2 (13.8 to 25)
Libya	Female	50 (33 to 70)	4.2 (2.9 to 5.8)	7 (5 to 10)	0.8 (0.6 to 1.1)	243 (171 to 327)	22.7 (16.1 to 30.6)	221 (140 to 319)	6.4 (4.2 to 9)	18 (13 to 25)	0.7 (0.5 to 1.1)	650 (437 to 920)	21.5 (15 to 30)
	Male	8 (6 to 11)	0.6 (0.4 to 0.8)	2 (2 to 3)	0.2 (0.1 to 0.3)	71 (52 to 99)	5.5 (4 to 7.6)	37 (26 to 51)	1 (0.7 to 1.4)	6 (5 to 8)	0.2 (0.2 to 0.3)	207 (152 to 276)	6.6 (4.9 to 8.7)
Morocco	Female	320 (214 to 427)	3.4 (2.5 to 4.4)	69 (52 to 90)	0.9 (0.7 to 1.4)	2411 (1808 to 3090)	27.8 (21.4 to 35.3)	1169 (753 to 1758)	6.3 (4.2 to 9.3)	140 (104 to 196)	0.9 (0.7 to 1.3)	4687 (3362 to 6519)	26.8 (19.6 to 36.3)
	Male	70 (48 to 93)	0.8 (0.6 to 1.1)	30 (20 to 39)	0.4 (0.3 to 0.6)	930 (648 to 1221)	11.5 (7.9 to 15.3)	293 (196 to 419)	1.7 (1.2 to 2.4)	86 (60 to 118)	0.6 (0.4 to 0.8)	2412 (1655 to 3300)	14.6 (10.2 to 19.8)
Oman	Female	12 (8 to 17)	2.8 (2 to 4)	2 (1 to 2)	0.6 (0.4 to 0.9)	58 (41 to 81)	16 (11.1 to 22)	74 (48 to 102)	6 (4.3 to 7.8)	4 (3 to 5)	0.6 (0.5 to 0.7)	155 (107 to 204)	16.3 (12.1 to 20.3)
	Male	4 (3 to 6)	0.6 (0.4 to 0.9)	1 (1 to 1)	0.3 (0.2 to 0.4)	34 (24 to 47)	6.4 (4.6 to 9.1)	37 (25 to 54)	1.7 (1.2 to 2.2)	3 (2 to 4)	0.4 (0.3 to 0.5)	129 (93 to 182)	8.9 (6.7 to 11.3)
Palestine	Female	21 (14 to 31)	3.7 (2.3 to 5.2)	5 (3 to 6)	1 (0.7 to 1.4)	127 (83 to 178)	24 (15.7 to 33)	91 (59 to 118)	5.6 (3.7 to 7)	11 (7 to 14)	1 (0.6 to 1.2)	323 (204 to 408)	23.5 (14.8 to 29.5)

	Male	4 (3 to 5)	0.8 (0.5 to 1.2)	1 (1 to 2)	0.4 (0.3 to 0.5)	39 (27 to 56)	8.9 (6 to 12.8)	19 (15 to 24)	1.2 (1 to 1.5)	4 (3 to 5)	0.4 (0.3 to 0.5)	121 (95 to 148)	9 (7.1 to 10.9)
Qatar	Female	2 (2 to 4)	3.4 (2.5 to 4.8)	0 (0 to 0)	0.9 (0.7 to 1.3)	10 (7 to 15)	20.7 (15.8 to 29.2)	30 (20 to 49)	6.7 (4.7 to 9.5)	1 (1 to 2)	1.1 (0.8 to 1.5)	52 (35 to 81)	20.5 (15 to 27.8)
	Male	1 (1 to 2)	1 (0.7 to 1.3)	0 (0 to 0)	0.5 (0.3 to 0.6)	11 (8 to 15)	10.3 (7.4 to 13.8)	29 (19 to 41)	1.8 (1.2 to 2.5)	2 (1 to 3)	0.4 (0.3 to 0.6)	86 (56 to 123)	8.6 (5.8 to 12)
Saudi Arabia	Female	104 (70 to 155)	2.6 (1.8 to 4.2)	19 (13 to 33)	0.8 (0.5 to 1.4)	634 (436 to 979)	19.2 (13.6 to 34.4)	1551 (886 to 2292)	10.2 (6.9 to 14.2)	60 (44 to 81)	0.8 (0.6 to 1.1)	2905 (1896 to 4176)	24.8 (18 to 32.9)
	Male	32 (22 to 47)	0.6 (0.4 to 0.9)	10 (7 to 15)	0.3 (0.2 to 0.4)	329 (231 to 475)	7.3 (5.2 to 11)	708 (491 to 1047)	3.2 (2.4 to 4.4)	61 (46 to 79)	0.6 (0.4 to 0.7)	2475 (1801 to 3381)	15 (11.4 to 19.2)
Sudan	Female	101 (54 to 160)	1.7 (1 to 2.6)	30 (18 to 43)	0.7 (0.4 to 1)	938 (523 to 1430)	17.4 (10.1 to 25.6)	418 (237 to 651)	3.1 (1.9 to 4.7)	58 (39 to 80)	0.7 (0.5 to 0.9)	1958 (1260 to 2903)	17.7 (11.7 to 24.7)
	Male	27 (17 to 50)	0.5 (0.3 to 0.9)	14 (9 to 25)	0.3 (0.2 to 0.5)	421 (264 to 775)	7.4 (4.6 to 13.7)	151 (91 to 227)	1.1 (0.7 to 1.7)	44 (28 to 63)	0.4 (0.3 to 0.6)	1377 (876 to 2029)	11.3 (7.2 to 16.6)
Syrian Arab Republic	Female	25 (15 to 34)	0.7 (0.4 to 1)	5 (3 to 7)	0.2 (0.1 to 0.3)	159 (96 to 213)	5.3 (3 to 7.2)	89 (52 to 124)	1.3 (0.7 to 1.7)	10 (5 to 14)	0.2 (0.1 to 0.3)	307 (175 to 423)	4.8 (2.7 to 6.5)
	Male	12 (9 to 17)	0.3 (0.2 to 0.5)	4 (3 to 6)	0.1 (0.1 to 0.2)	138 (97 to 187)	4 (2.7 to 5.5)	49 (32 to 70)	0.7 (0.4 to 1)	11 (7 to 15)	0.2 (0.1 to 0.2)	312 (207 to 437)	4.5 (3 to 6.2)
Tunisia	Female	94 (70 to 123)	2.9 (2.3 to 3.8)	13 (10 to 18)	0.5 (0.4 to 0.8)	429 (337 to 555)	14.7 (11.6 to 19.3)	376 (248 to 559)	5.6 (3.7 to 8.3)	29 (20 to 42)	0.5 (0.3 to 0.7)	925 (636 to 1323)	14.1 (9.7 to 20)

	Male	22 (17 to 28)	0.7 (0.5 to 0.9)	7 (5 to 9)	0.3 (0.2 to 0.4)	200 (155 to 256)	6.8 (5.2 to 8.9)	118 (78 to 168)	1.8 (1.2 to 2.6)	20 (14 to 29)	0.3 (0.2 to 0.5)	572 (385 to 809)	9.1 (6.2 to 12.8)
Turkey	Female	643 (420 to 876)	3 (2 to 4)	165 (116 to 212)	0.9 (0.6 to 1.2)	4563 (3097 to 6051)	22.6 (15.5 to 29.6)	2141 (1597 to 2881)	4.6 (3.4 to 6.1)	261 (200 to 342)	0.6 (0.4 to 0.8)	6544 (4951 to 8494)	14.1 (10.7 to 18.2)
	Male	249 (176 to 372)	1.2 (0.8 to 1.8)	92 (68 to 137)	0.5 (0.4 to 0.8)	2895 (2089 to 4322)	14.3 (10.4 to 21.4)	1130 (837 to 1599)	2.5 (1.8 to 3.5)	193 (145 to 293)	0.5 (0.4 to 0.7)	5526 (4158 to 8042)	12.6 (9.6 to 18.5)
United Arab Emirates	Female	11 (7 to 17)	3.5 (1.9 to 5.5)	1 (1 to 2)	0.9 (0.4 to 1.6)	54 (32 to 81)	23.6 (11.3 to 37.6)	130 (67 to 207)	4.9 (2.6 to 7.7)	7 (4 to 12)	0.7 (0.3 to 1.1)	343 (174 to 544)	18.7 (9.3 to 30)
	Male	12 (7 to 19)	1.6 (0.8 to 2.4)	2 (1 to 4)	0.7 (0.3 to 1.1)	103 (60 to 159)	17.5 (8.2 to 27.8)	314 (147 to 522)	3.5 (1.7 to 5.6)	35 (16 to 55)	0.9 (0.4 to 1.4)	1531 (716 to 2473)	24 (11.7 to 37.3)
Yemen	Female	55 (29 to 92)	1.7 (1 to 2.7)	17 (10 to 26)	0.7 (0.4 to 1)	517 (298 to 806)	17.3 (10.3 to 26.5)	283 (167 to 434)	2.9 (1.8 to 4.3)	49 (33 to 68)	0.7 (0.5 to 1)	1606 (1029 to 2365)	19.2 (12.9 to 27.3)
	Male	14 (8 to 25)	0.5 (0.3 to 0.8)	7 (4 to 12)	0.3 (0.2 to 0.5)	216 (129 to 395)	7.1 (4.3 to 13.1)	73 (46 to 114)	0.8 (0.5 to 1.2)	23 (15 to 36)	0.3 (0.2 to 0.5)	752 (490 to 1169)	9 (5.9 to 14.1)

Table S2. Age-standardized death and disability-adjusted life years (DALYs) percent and rate of thyroid cancer attributable to high body mass index (BMI) in North Africa and Middle East region in 1990 and 2019, by sex and country.

Location	Sex	1990				2019			
		Deaths		DALYs (Disability-Adjusted Life Years)		Deaths		DALYs (Disability-Adjusted Life Years)	
		Percent	Rate	Percent	Rate	Percent	Rate	Percent	Rate
**North Africa and Middle East**	**Both**	**11.2 (5.7 to 18.6)**	**0.1 (0.0 to 0.1)**	**11.3 (5.8 to 18.6)**	**1.6 (0.8 to 2.7)**	**16.7 (9.1 to 25.8)**	**0.1 (0.0 to 0.1)**	**17.1 (9.3 to 26.1)**	**2.5 (1.4 to 4.0)**
	**Female**	**10.9 (5.8 to 17.9)**	**0.1 (0.0 to 0.1)**	**10.9 (5.8 to 17.6)**	**2.1 (1.1 to 3.5)**	**15.0 (8.6 to 22.8)**	**0.1 (0.1 to 0.2)**	**15.1 (8.6 to 22.8)**	**2.7 (1.5 to 4.3)**
	**Male**	**11.8 (3.3 to 24.0)**	**0.0 (0.0 to 0.1)**	**12.0 (3.5 to 24.0)**	**1.2 (0.3 to 2.5)**	**19.2 (6.2 to 35.2)**	**0.1 (0.0 to 0.2)**	**19.9 (6.6 to 35.8)**	**2.4 (0.8 to 4.3)**
Afghanistan	Both	6.4 (2.8 to 11.9)	0.1 (0.0 to 0.1)	6.5 (2.9 to 11.9)	1.5 (0.5 to 3.1)	10.2 (5.4 to 16.5)	0.1 (0.0 to 0.2)	10.1 (5.4 to 16.5)	2.4 (1.1 to 4.5)
	Female	6.6 (3.0 to 11.9)	0.1 (0.0 to 0.2)	6.6 (3.0 to 12.0)	2.2 (0.7 to 4.9)	9.7 (5.2 to 15.7)	0.1 (0.1 to 0.2)	9.6 (5.3 to 15.6)	3.4 (1.3 to 6.7)
	Male	6.2 (1.5 to 14.5)	0.0 (0.1 to 0.1)	6.2 (1.5 to 14.7)	0.7 (0.1 to 1.9)	11.4 (3.3 to 22.8)	0.1 (0.0 to 0.1)	11.3 (3.3 to 22.5)	1.5 (0.4 to 3.4)
Algeria	Both	10.2 (4.9 to 17.0)	0.1 (0.0 to 0.1)	10.4 (5.3 to 16.9)	1.4 (0.7 to 2.5)	16.2 (8.8 to 25.1)	0.1 (0.0 to 0.1)	16.2 (8.9 to 24.7)	2.4 (1.3 to 4.1)
	Female	10.1 (5.3 to 16.6)	0.1 (0.0 to 0.1)	10.4 (5.5 to 16.7)	2.1 (1.0 to 3.6)	15.0 (8.4 to 22.8)	0.1 (0.1 to 0.2)	15.0 (8.5 to 22.9)	3.1 (1.7 to 5.4)
	Male	10.0 (2.6 to 21.6)	0.0 (0.0 to 0.1)	10.2 (2.7 to 21.5)	0.8 (0.2 to 1.8)	18.1 (5.8 to 34.3)	0.1 (0.0 to 0.1)	18.4 (5.9 to 34.2)	1.8 (0.6 to 3.6)
Bahrain	Both	15.1 (8.1 to 23.5)	0.1 (0.1 to 0.2)	15.8 (8.5 to 24.5)	2.7 (1.4 to 4.4)	18.6 (10.3 to 27.8)	0.1 (0.1 to 0.2)	19.4 (11.0 to 29.0)	2.8 (1.5 to 4.5)
	Female	14.6 (8.0 to 22.6)	0.2 (0.1 to 0.3)	15.2 (8.5 to 23.1)	3.7 (1.9 to 6.1)	17.5 (10.0 to 25.9)	0.2 (0.1 to 0.3)	18.0 (10.5 to 26.6)	4.2 (2.2 to 6.8)
	Male	16..5 (5.1 to 31.8)	0.1 (0.0 to 0.2)	17.1 (5.3 to 32.4)	1.8 (0.6 to 3.6)	21.7 (7.0 to 39.2)	0.1 (0.0 to 0.2)	22.3 (7.3 to 39.8)	1.9 (0.6 to 3.6)
Egypt	Both	12.2 (6.2 to 19.9)	0.0 (0.0 to 0.1)	11.2 (5.5 to 18.2)	1.4 (0.7 to 2.5)	19.4 (10.6 to 29.4)	0.1 (0.0 to 0.2)	19.4 (10.6 to 29.4)	2.4 (1.1 to 4.3)
	Female	12.1 (6.4 to 19.2)	0.1 (0.0 to 0.1)	11.1 (5.7 to 17.8)	1.8 (0.9 to 3.4)	17.7 (10.2 to 26.3)	0.1 (0.0 to 0.2)	17.7 (10.2 to 26.1)	2.6 (1.3 to 4.5)
	Male	12.3 (3.4 to 25.2)	0.0 (0.0 to 0.1)	11.4 (3.2 to 23.4)	1.0 (0.3 to 2.1)	21.3 (6.9 to 38.4)	0.1 (0.0 to 0.2)	21.3 (7.1 to 37.9)	2.3 (0.7 to 4.6)
Iran (Islamic Republic of)	Both	9.0 (4.5 to 15.2)	0.0 (0.0 to 0.1)	9.4 (4.6 to 15.7)	1.0 (0.5 to 1.7)	14.4 (7.8 to 22.7)	0.1 (0.0 to 0.1)	14.6 (7.7 to 22.9)	1.9 (1.0 to 3.1)
	Female	9.0 (4.8 to 14.8)	0.0 (0.0 to 0.1)	9.4 (5.0 to 15.4)	1.3 (0.6 to 2.2)	13.0 (7.5 to 20.1)	0.1 (0.0 to 0.1)	13.0 (7.5 to 20.3)	2.0 (1.0 to 3.2)
	Male	9.3 (2.5 to 19.7)	0.0 (0.0 to 0.1)	9.4 (2.5 to 19.8)	0.7 (0.2 to 1.6)	16.4 (5.1 to 30.8)	0.1 (0.0 to 0.1)	16.6 (5.1 to 31.0)	1.8 (0.5 to 3.5)

Iraq	Both	15.2 (7.4 to 24.5)	0.1 (0.0 to 0.1)	15.4 (7.6 to 24.4)	2.3 (1.0 to 3.9)	17.3 (8.7 to 27.3)	0.1 (0.1 to 0.2)	17.4 (9.0 to 27.3)	3.3 (1.6 to 5.6)
	Female	13.5 (7.0 to 21.1)	0.1 (0.0 to 0.2)	13.5 (7.2 to 20.9)	2.4 (1.1 to 4.4)	14.5 (8.2 to 22.5)	0.1 (0.1 to 0.2)	14.6 (8.2 to 22.6)	3.1 (1.6 to 5.2)
	Male	17.7 (5.0 to 34.3)	0.1 (0.0 to 0.2)	18.1 (5.3 to 34.8)	2.1 (0.6 to 4.3)	20.5 (6.5 to 37.8)	0.1 (0.0 to 0.3)	20.9 (6.7 to 38.3)	3.6 (1.0 to 6.8)
Jordan	Both	14.6 (7.8 to 23.1)	0.1 (0.1 to 0.2)	15.0 (8.0 to 23.2)	3.1 (1.6 to 5.1)	21.1 (11.8 to 31.4)	0.1 (0.1 to 0.2)	21.5 (12.1 to 31.7)	3.1 (1.7 to 4.8)
	Female	14.0 (7.8 to 21.7)	0.2 (0.1 to 0.3)	14.2 (8.0 to 22.0)	4.1 (2.2 to 6.8)	18.7 (10.8 to 27.6)	0.1 (0.1 to 0.2)	18.9 (11.0 to 27.8)	3.5 (2.0 to 5.8)
	Male	16.2 (4.9 to 31.3)	0.1 (0.0 to 0.2)	16.6 (5.0 to 32.0)	2.1 (0.6 to 4.1)	25.0 (8.5 to 43.6)	0.1 (0.0 to 0.2)	25.4 (8.6 to 43.8)	2.7 (0.9 to 4.9)
Kuwait	Both	16.8 (9.1 to 25.4)	0.1 (0.1 to 0.2)	17.7 (9.6 to 26.6)	2.8 (1.5 to 4.4)	22.9 (12.1 to 34.8)	0.1 (0.1 to 0.2)	23.7 (12.9 to 35.4)	3.0 (1.6 to 4.7)
	Female	16.3 (9.2 to 24.6)	0.2 (0.1 to 0.3)	16.9 (9.7 to 25.4)	4.6 (2.6 to 7.0)	20.4 (12.1 to 29.6)	0.1 (0.1 to 0.2)	21.2 (12.6 to 30.2)	3.1 (1.8 to 4.8)
	Male	18.6 (5.8 to 34.9)	0.1 (0.0 to 0.1)	19.6 (6.1 to 36.3)	1.8 (0.5 to 3.4)	24.9 (8.4 to 43.8)	0.1 (0.0 to 0.2)	26.0 (9.0 to 44.7)	2.9 (1.0 to 5.2)
Lebanon	Both	12.4 (6.2 to 20.4)	0.1 (0.0 to 0.2)	12.5 (6.2 to 20.6)	2.4 (1.1 to 4.2)	17.8 (9.1 to 28.1)	0.1 (0.1 to 0.2)	17.8 (9.4 to 27.9)	3.6 (1.8 to 5.9)
	Female	11.8 (6.1 to 19.3)	0.1 (0.0 to 0.2)	11.8 (6.0 to 18.9)	2.9 (1.4 to 5.1)	15.6 (8.8 to 23.7)	0.1 (0.1 to 0.2)	15.4 (8.8 to 23.5)	3.3 (1.7 to 5.7)
	Male	13.5 (3.9 to 26.7)	0.1 (0.0 to 0.1)	14.0 (4.1 to 27.8)	1.9 (0.5 to 4.0)	20.2 (6.2 to 37.1)	0.1 (0.0 to 0.3)	20.9 (6.6 to 38.0)	4.0 (1.2 to 7.7)
Libya	Both	14.1 (7.6 to 21.7)	0.1 (0.0 to 0.1)	14.3 (7.8 to 22.3)	1.9 (1.0 to 3.2)	18.4 (10.2 to 27.2)	0.1 (0.0 to 0.1)	18.5 (10.5 to 27.1)	2.6 (1.4 to 4.2)
	Female	13.8 (7.6 to 21.9)	0.1 (0.1 to 0.2)	14.0 (7.7 to 22.0)	3.2 (1.6 to 5.7)	17.2 (10.0 to 25.8)	0.1 (0.1 to 0.2)	17.3 (10.1 to 25.8)	3.7 (1.9 to 6.3)
	Male	15.1 (4.4 to 29.6)	0.0 (0.0 to 0.1)	15.5 (4.7 to 30.2)	0.9 (0.3 to 1.8)	21.6 (6.9 to 39.3)	0.1 (0.0 to 0.1)	22.0 (7.2 to 39.4)	1.5 (0.5 to 2.7)
Morocco	Both	9.3 (4.5 to 16.0)	0.1 (0.0 to 0.1)	9.4 (4.7 to 15.8)	1.9 (0.9 to 3.3)	15.0 (7.7 to 24.0)	0.1 (0.1 to 0.2)	15.1 (8.0 to 23.7)	3.1 (1.6 to 5.3)
	Female	8.9 (4.5 to 15.2)	0.1 (0.0 to 0.2)	9.0 (4.5 to 15.3)	2.5 (1.2 to 4.5)	14.1 (7.7 to 22.2)	0.1 (0.1 to 0.2)	14.1 (7.8 to 22.0)	3.8 (1.9 to 6.6)
	Male	10.1 (2.8 to 21.1)	0.1 (0.0 to 0.1)	10.4 (2.9 to 21.4)	1.2 (0.3 to 2.6)	16.4 (4.8 to 31.7)	0.1 (0.0 to 0.2)	16.8 (4.9 to 32.3)	2.5 (0.7 to 4.9)
Oman	Both	7.6 (3.6 to 13.4)	0.1 (0.0 to 0.1)	8.4 (4.0 to 14.4)	0.9 (0.4 to 1.6)	18.8 (10.2 to 28.9)	0.1 (0.0 to 0.1)	19.5 (10.8 to 29.6)	2.3 (1.2 to 3.7)
	Female	7.8 (3.8 to 13.4)	0.0 (0.0 to 0.1)	8.5 (4.3 to 14.3)	1.4 (0.6 to 2.5)	17.1 (9.8 to 25.7)	0.1 (0.1 to 0.2)	17.6 (10.2 to 26.2)	2.9 (1.6 to 4.6)
	Male	7.8 (2.0 to 16.9)	0.0 (0.0 to 0.0)	8.5 (2.2 to 18.1)	0.5 (0.1 to 1.3)	21.1 (6.8 to 38.7)	0.1 (0.0 to 0.2)	22.0 (7.0 to 39.6)	2.0 (0.6 to 3.8)
Palestine	Both	9.4 (4.3 to 16.2)	0.1 (0.0 to 0.1)	10.6 (5.2 to 17.7)	1.8 (0.8 to 3.2)	12.3 (6.4 to 19.6)	0.1 (0.0 to 0.2)	13.5 (7.2 to 21.4)	2.2 (1.2 to 3.7)

	Female	9.3 (4.4 to 16.0)	0.1 (0.0 to 0.2)	10.4 (5.1 to 17.3)	2.5 (1.1 to 4.6)	11.9 (6.1 to 19.2)	0.1 (0.1 to 0.2)	12.8 (7.0 to 20.0)	3.0 (1.5 to 5.1)
	Male	9.9 (2.6 to 21.7)	0.0 (0.0 to 0.1)	11.0 (3.0 to 23.3)	1.0 (0.2 to 2.2)	14.0 (4.1 to 27.8)	0.1 (0.0 to 0.1)	15.8 (4.7 to 30.3)	1.4 (0.4 to 2.8)
Qatar	Both	17.3 (9.3 to 27.0)	0.1 (0.1 to 0.2)	18.4 (9.7 to 28.4)	2.6 (1.3 to 4.4)	25.0 (13.2 to 37.8)	0.1 (0.1 to 0.2)	25.5 (13.7 to 38.4)	2.9 (1.5 to 4.9)
	Female	16.4 (9.4 to 24.8)	0.2 (0.1 to 0.3)	17.2 (9.9 to 25.7)	3.6 (1.9 to 5.9)	21.0 (12.2 to 30.0)	0.2 (0.1 to 0.4)	21.6 (12.9 to 30.9)	4.4 (2.5 to 7.1)
	Male	19.2 (5.9 to 35.8)	0.1 (0.0 to 0.2)	20.1 (6.3 to 37.3)	2.1 (0.6 to 4.3)	28.1 (9.9 to 47.7)	0.1 (0.0 to 0.2)	28.5 (10.2 to 47.9)	2.5 (0.8 to 4.5)
Saudi Arabia	Both	12.0 (6.2 to 19.8)	0.1 (0.0 to 0.1)	12.6 (6.6 to 20.6)	1.5 (0.7 to 2.8)	22.6 (12.4 to 34.1)	0.1 (0.1 to 0.2)	23.1 (12.9 to 34.0)	4.4 (2.2 to 6.8)
	Female	11.7 (6.1 to 19.2)	0.1 (0.0 to 0.2)	12.3 (6.5 to 19.7)	2.4 (1.1 to 4.6)	20.4 (12.1 to 29.7)	0.2 (0.1 to 0.3)	20.6 (12.3 to 29.7)	5.1 (2.8 to 8.5)
	Male	12.7 (3.8 to 25.3)	0.0 (0.0 to 0.1)	13.5 (4.0 to 26.7)	1.0 (0.3 to 2.2)	24.8 (8.3 to 43.3)	0.1 (0.0 to 0.2)	25.7 (8.7 to 44.4)	3.9 (1.3 to 6.9)
Sudan	Both	6.5 (2.8 to 12.0)	0.0 (0.0 to 0.1)	6.8 (3.0 to 12.5)	0.8 (0.3 to 1.7)	13.7 (6.9 to 22.4)	0.1 (0.0 to 0.1)	14.0 (7.2 to 22.7)	2.0 (0.9 to 3.5)
	Female	6.7 (3.2 to 12.1)	0.0 (0.0 to 0.1)	7.0 (3.4 to 12.5)	1.2 (0.5 to 2.5)	12.4 (6.8 to 19.7)	0.1 (0.0 to 0.1)	12.8 (7.1 to 20.1)	2.3 (1.1 to 4.0)
	Male	6.1 (1.4 to 14.4)	0.0 (0.0 to 0.0)	6.4 (1.5 to 15.0)	0.5 (0.1 to 1.2)	15.4 (4.4 to 29.6)	0.1 (0.0 to 0.1)	15.8 (4.6 to 30.1)	1.8 (0.5 to 3.7)
Syrian Arab Republic	Both	11.8 (5.6 to 20.0)	0.0 (0.0 to 0.0)	12.1 (5.7 to 20.4)	0.6 (0.2 to 1.0)	15.9 (8.0 to 25.8)	0.0 (0.0 to 0.1)	15.8 (8.1 to 25.4)	0.7 (0.3 to 1.3)
	Female	11.7 (6.0 to 19.0)	0.0 (0.0 to 0.0)	11.9 (6.2 to 19.2)	0.6 (0.3 to 1.1)	14.4 (8.0 to 22.6)	0.0 (0.0 to 0.0)	14.2 (8.0 to 22.2)	0.7 (0.3 to 1.2)
	Male	12.1 (3.2 to 24.7)	0.0 (0.0 to 0.0)	12.2 (3.3 to 24.8)	0.5 (0.1 to 1.0)	17.4 (5.2 to 33.0)	0.0 (0.0 to 0.1)	17.5 (5.2 to 32.9)	0.8 (0.2 to 1.6)
Tunisia	Both	10.9 (5.4 to 18.4)	0.0 (0.0 to 0.1)	11.2 (5.8 to 18.7)	1.2 (0.6 to 2.1)	15.9 (8.2 to 25.4)	0.1 (0.0 to 0.1)	16.1 (8.5 to 25.2)	1.9 (0.9 to 3.2)
	Female	10.7 (5.5 to 17.3)	0.1 (0.0 to 0.1)	10.9 (5.7 to 17.7)	1.6 (0.8 to 2.8)	14.6 (8.0 to 23.0)	0.1 (0.0 to 0.1)	14.7 (8.0 to 23.1)	2.1 (1.0 to 3.6)
	Male	11.3 (3.1 to 23.5)	0.0 (0.0 to 0.1)	11.8 (3.3 to 23.9)	0.8 (0.2 to 1.7)	17.7 (5.3 to 33.7)	0.1 (0.0 to 0.1)	18.3 (5.7 to 34.5)	1.7 (0.5 to 3.2)
Turkey	Both	13.6 (7.1 to 22.1)	0.1 (0.1 to 0.2)	14.0 (7.2 to 22.6)	2.6 (1.3 to 4.5)	18.8 (10.1 to 28.9)	0.1 (0.0 to 0.2)	19.4 (10.4 to 29.6)	2.6 (1.3 to 4.5)
	Female	13.5 (7.3 to 21.3)	0.1 (0.1 to 0.2)	13.7 (7.5 to 21.7)	3.1 (1.5 to 5.4)	17.1 (9.6 to 26.1)	0.1 (0.1 to 0.2)	17.5 (9.8 to 26.4)	2.5 (1.3 to 4.2)
	Male	14.0 (3.9 to 27.9)	0.1 (0.0 to 0.2)	14.3 (4.1 to 28.3)	2.1 (0.6 to 4.6)	21.1 (6.6 to 38.8)	0.1 (0.0 to 0.2)	21.7 (6.9 to 39.5)	2.7 (0.9 to 5.4)
United Arab Emirates	Both	16.5 (8.3 to 27.1)	0.1 (0.0 to 0.3)	17.5 (8.7 to 28.8)	3.5 (1.2 to 6.7)	26.6 (12.1 to 42.5)	0.2 (0.1 to 0.4)	27.3 (12.1 to 43.3)	6.2 (2.1 to 12.2)
	Female	14.4 (8.4 to 21.9)	0.1 (0.0 to 0.3)	15.1 (8.7 to 22.8)	3.6 (1.3 to 7.0)	20.4 (12.6 to 29.4)	0.1 (0.1 to 0.3)	20.8 (12.9 to 29.5)	3.9 (1.6 to 7.6)

	Male	18.2 (5.7 to 34.0)	0.1 (0.0 to 0.3)	19.1 (6.1 to 35.4)	3.4 (0.8 to 7.7)	28.4 (10.0 to 47.7)	0.2 (0.1 to 0.5)	29.1 (10.4 to 48.4)	7.0 (1.9 to 14.6)
Yemen	Both	4.2 (1.5 to 8.4)	0.0 (0.0 to 0.0)	4.4 (1.6 to 8.7)	0.5 (0.2 to 1.2)	7.2 (3.4 to 12.5)	0.0 (0.0 to 0.1)	7.7 (3.7 to 13.0)	1.1 (0.5 to 2.0)
	Female	4.3 (1.7 to 8.4)	0.0 (0.0 to 0.1)	4.5 (1.8 to 8.7)	0.8 (0.3 to 1.7)	7.0 (3.5 to 12.0)	0.1 (0.0 to 0.1)	7.4 (3.7 to 12.5)	1.4 (0.6 to 2.7)
	Male	4.0 (0.7 to 10.4)	0.0 (0.0 to 0.0)	4.2 (0.8 to 10.9)	0.3 (0.1 to 0.9)	7.7 (2.1 to 16.7)	0.0 (0.0 to 0.1)	8.1 (2.2 to 17.4)	0.7 (0.2 to 1.7)

Table S3. Decomposition analysis of thyroid cancer incidence in North Africa and Middle East region over 1990-2019, by sex and country.

Location	Sex	New cases		Expected new cases in 2019		% 1990 - 2019 new cases change cause			% 1990 - 2019 new cases overall change
		Year		Given population growth alone	Given population growth and aging	Due to population growth	Due to population aging	Due to change in incidence rates	
		1990	2019						
**North Africa and Middle East**	**Female**	**2970**	**13586**	**5163**	**6959**	**73.8%**	**60.5%**	**223.1%**	**357.4%**
	**Male**	**911**	**5667**	**1631**	**2231**	**78.9%**	**65.8%**	**377.1%**	**521.8%**
Afghanistan	Female	100	364	323	260	223%	-63.1%	104.4%	264.3%
	Male	22	75	78	50	247.7%	-122.2%	110.4%	235.9%
Algeria	Female	312	1484	516	755	65.2%	76.4%	233.4%	375.0%
	Male	62	343	102	151	65.8%	79.4%	310.3%	455.6%
Bahrain	Female	4	32	9	17	155.2%	214.9%	417.4%	787.6%
	Male	1	14	4	8	204.8%	273.7%	464.7%	943.2%
Egypt	Female	434	1329	761	831	75.5%	16.2%	114.9%	206.6%
	Male	141	679	253	293	80.2%	28.3%	274.5%	382.9%
Iran (Islamic Republic of)	Female	400	2242	580	1035	44.9%	113.6%	301.6%	460.1%
	Male	139	956	199	334	43.1%	97.6%	447.7%	588.5%
Iraq	Female	138	915	330	431	138.4%	72.6%	349.7%	560.8%
	Male	51	367	121	157	140.3%	70.4%	415.7%	626.3%
Jordan	Female	46	229	138	197	201.6%	128.1%	70.4%	400.1%
	Male	11	93	36	53	214.6%	153.6%	345.0%	713.2%
Kuwait	Female	28	95	77	127	173.6%	181.2%	-115.7%	239.1%
	Male	9	54	21	30	135.2%	105.8%	282.6%	523.7%
Lebanon	Female	67	308	109	144	62.2%	51.3%	244.1%	357.7%
	Male	19	123	29	37	54.0%	43.5%	457.3%	554.8%
Libya	Female	50	221	81	145	61.1%	127.3%	150.7%	339.0%
	Male	8	37	13	19	57.0%	86.0%	222.3%	365.4%
Morocco	Female	320	1169	451	620	40.9%	52.8%	171.2%	264.9%
	Male	70	293	100	144	43.4%	61.9%	213.0%	318.3%
Oman	Female	12	74	24	35	101.3%	85.6%	325.7%	512.6%
	Male	4	37	11	14	160.6%	73.2%	558.4%	792.2%
Palestine	Female	21	91	51	59	136.7%	39.5%	151.6%	327.8%
	Male	4	19	9	12	142.1%	76.9%	183.7%	402.8%
Qatar	Female	2	30	12	18	392.2%	213.5%	503.6%	1109.2%
	Male	1	29	10	13	618.7%	199.3%	1137.1%	1955.1%
Saudi Arabia	Female	104	1551	218	361	110.2%	137.5%	1147.9%	1395.6%
	Male	32	708	75	111	132.5%	109.3%	1846%	2087.7%
Sudan	Female	101	418	203	221	101.3%	18.4%	196.2%	315.9%
	Male	27	151	55	59	102.8%	16.1%	340.0%	458.9%
Syrian Arab Republic	Female	25	89	29	51	18.0%	90.7%	152.6%	261.4%
	Male	12	49	13	24	7.0%	86.6%	208.4%	302.0%
Tunisia	Female	94	376	131	195	39.6%	67.8%	193.0%	300.4%
	Male	22	118	29	45	34.7%	70.2%	336.8%	441.7%
Turkey	Female	643	2141	878	1404	36.6%	81.8%	114.6%	233.0%

	Male	249	1130	338	538	35.6%	80.2%	237.0%	352.8%
United Arab Emirates	Female	11	130	43	79	282.9%	322.4%	451.2%	1056.5%
	Male	12	314	68	110	454.1%	344.4%	1665.6%	2464.0%
Yemen	Female	55	283	128	157	131.2%	52.9%	228.1%	412.2%
	Male	14	73	32	39	127.7%	50.9%	242.7%	421.4%
